# The immune-endothelial axis in neurovascular pathophysiology: a framework for targeted interventions

**DOI:** 10.3389/fimmu.2026.1809878

**Published:** 2026-05-18

**Authors:** Chang Li, Baofeng Xu, Rui Liu

**Affiliations:** 1 Department of Very Important People, China-Japan Union Hospital of Jilin University, Changchun, Jilin, China; 2 Department of Stroke Center, First Hospital of Jilin University, Changchun, Jilin, China; 3 Hunan Provincial University Key Laboratory of the Fundamental and Clinical Research on Neurodegenerative Diseases, Changsha Medical University, Changsha, Hunan, China

**Keywords:** blood-brain barrier, cerebrovascular disorder, endothelial dysfunction, immune-endothelial interactions, neurodegenerative disease, neuroimmune signaling, neuroinflammation, vascular aging

## Abstract

The cerebrovascular endothelium has been reconceptualized from a mere passive barrier to an active regulator of neuroimmune responses. The interaction between immune cells and endothelial cells, particularly at the blood-brain barrier, is pivotal in the context of neuroinflammation and vascular dysfunction associated with various neurological disorders. This comprehensive review synthesizes recent advancements in the molecular and cellular mechanisms that govern this interaction. We investigate how activated endothelial cells interact with immune cells through adhesion pathways, cytokine networks, and metabolic cross-talk, thereby contributing to the pathogenesis of disease. Special attention is given to the influence of vascular aging in exacerbating this dysregulation. Additionally, we assess the translational implications of our findings, including the identification of novel biomarkers, advancements in neuroimaging techniques, and therapeutic strategies aimed at restoring barrier integrity and modulating neuroimmune responses. A comprehensive understanding of the immune-endothelial axis provides a crucial framework for elucidating neurovascular pathophysiology and informing targeted interventions.

## 1 Introduction

The central nervous system (CNS) represents the most complex organ system within the human body, playing a pivotal role in cognition, behavior, and the maintenance of physiological homeostasis. The functional integrity of the CNS is critically dependent on a meticulously regulated microenvironment, which is predominantly protected by the highly specialized blood-brain barrier (BBB) (1). Serving as the essential interface between the circulatory system and the brain parenchyma, the BBB is not merely a static barrier; rather, it is a dynamic, multicellular structure that effectively safeguards sensitive neural tissue (2). For decades, the CNS was regarded as an immunologically privileged site (3). However, this perspective has been fundamentally revised. Neuroinflammation—characterized by the activation of resident CNS glial cells and the recruitment and actions of peripheral immune cells—is now acknowledged as a central pathological feature across a broad range of neurological disorders (4). From acute events like ischemic stroke to chronic neurodegenerative diseases such as Alzheimer’s disease (AD) and multiple sclerosis (MS), as well as traumatic injuries and infections, a dysregulated immune response within the CNS is recognized as be a key driver of disease initiation and progression, rather than merely a bystander effect (5, 6).

At the core of this neuro-immune interaction is the communication between the immune system and the cerebrovascular endothelium. Endothelial cells forming the BBB are not merely passive targets of inflammation; rather, they actively participate in the immune response (7). Under pathological conditions, these cells become “activated,” leading to the upregulation of adhesion molecules such as intercellular adhesion molecule-1 (ICAM-1) and vascular cell adhesion molecule-1 (VCAM-1), the secretion of chemokines, and both functional and structural changes that compromise BBB integrity (8). This endothelial dysfunction is increasingly recognized not simply as a consequence of neuroinflammation but as a pivotal early event that initiates a detrimental cycle: immune signaling disrupts the BBB, facilitating further infiltration of peripheral immune cells, which subsequently release additional inflammatory mediators, thereby perpetuating neuroinflammation and exacerbating neuronal damage (9, 10). Recent advancements in single-cell and spatial transcriptomics have revealed the unexpected heterogeneity of brain endothelial cells, identifying distinct inflammatory signatures (11, 12). Simultaneously, the development of sophisticated models, including human induced pluripotent stem cell (hiPSC)-derived brain organoids and microfluidic BBB-on-a-chip systems, offers powerful tools for deciphering human-specific pathophysiological mechanisms and identifying intrinsic defects within endothelial cells themselves (13, 14).

Although the significance of immune-endothelial interactions is well recognized, the specific molecular mechanisms governing this axis—spanning processes such as leukocyte migration, cytokine signaling, metabolic reprogramming, and the effects of aging—remain inadequately elucidated. Current reviews frequently fall short in integrating recent technological advancements and in linking the mechanisms of immune-endothelial crosstalk to their implications in acute, chronic, and autoimmune neurological disorders. This review seeks to address this critical gap by providing a comprehensive analysis of the current understanding of immune-endothelial interactions within these diseases. This review will systematically elucidate the central role of the immune-endothelial interface in neurological disorders, focusing on: deconstructing its key cellular and molecular mechanisms; clarifying the regulatory impact of aging on the immune-endothelial axis; revealing the differential roles of this interaction in acute, chronic, and autoimmune neurological diseases; evaluating innovative diagnostic strategies based on this axis; and prospecting novel therapeutic pathways targeting this interface. By integrating multi-dimensional evidence, this article aims to provide a systematic framework for understanding the interactive mechanisms of neuroinflammation and vascular dysfunction and to promote the development of related precision diagnostic and therapeutic strategies.

To ensure methodological transparency, a systematic literature search was performed in the PubMed, Web of Science, and Scopus databases for articles published between January 2000 and February 2026. The search strategy combined controlled vocabulary (MeSH terms in PubMed, Emtree terms in Scopus) and free-text keywords, including, but not limited to: immune-endothelial interactions, blood-brain barrier(BBB), neuroinflammation, adhesion molecules, cytokine networks, chemokines, antigen presentation, stroke, Alzheimer’s disease(AD), multiple sclerosis(MS), traumatic brain injury(TBI), vascular aging, cellular senescence, mitochondrial dysfunction, biomarkers, neuroimaging, organ-on-a-chip, and therapeutic targeting. Additional studies were identified by manual screening of reference lists from included articles and relevant reviews. Inclusion criteria were as follows (1): peer-reviewed original research, reviews, or clinical studies; (2) articles published in English; (3) studies investigating immune-endothelial interactions in the context of neurological disorders, specifically stroke, Alzheimer’s disease, multiple sclerosis, or traumatic brain injury; and (4) studies reporting molecular mechanisms, cellular interactions, or translational insights relevant to the immune-endothelial axis. Exclusion criteria were: (1) non-English publications; (2) conference abstracts, editorials, or commentaries without full text; (3) studies focused exclusively on peripheral vascular beds without relevance to the central nervous system; and (4) studies addressing neurological disorders without direct investigation of immune-endothelial crosstalk. Consistent with the narrative scope of this review, no formal quality assessment or quantitative synthesis (e.g., meta-analysis) was performed; the PRISMA guidelines were consulted as a reference for reporting transparency but were not applied as a systematic review methodology (15, 16). Instead, findings were synthesized thematically to provide a comprehensive overview of the field.

## 2 Key cellular and molecular mechanisms in immune-endothelial interactions

**Figure 1:**
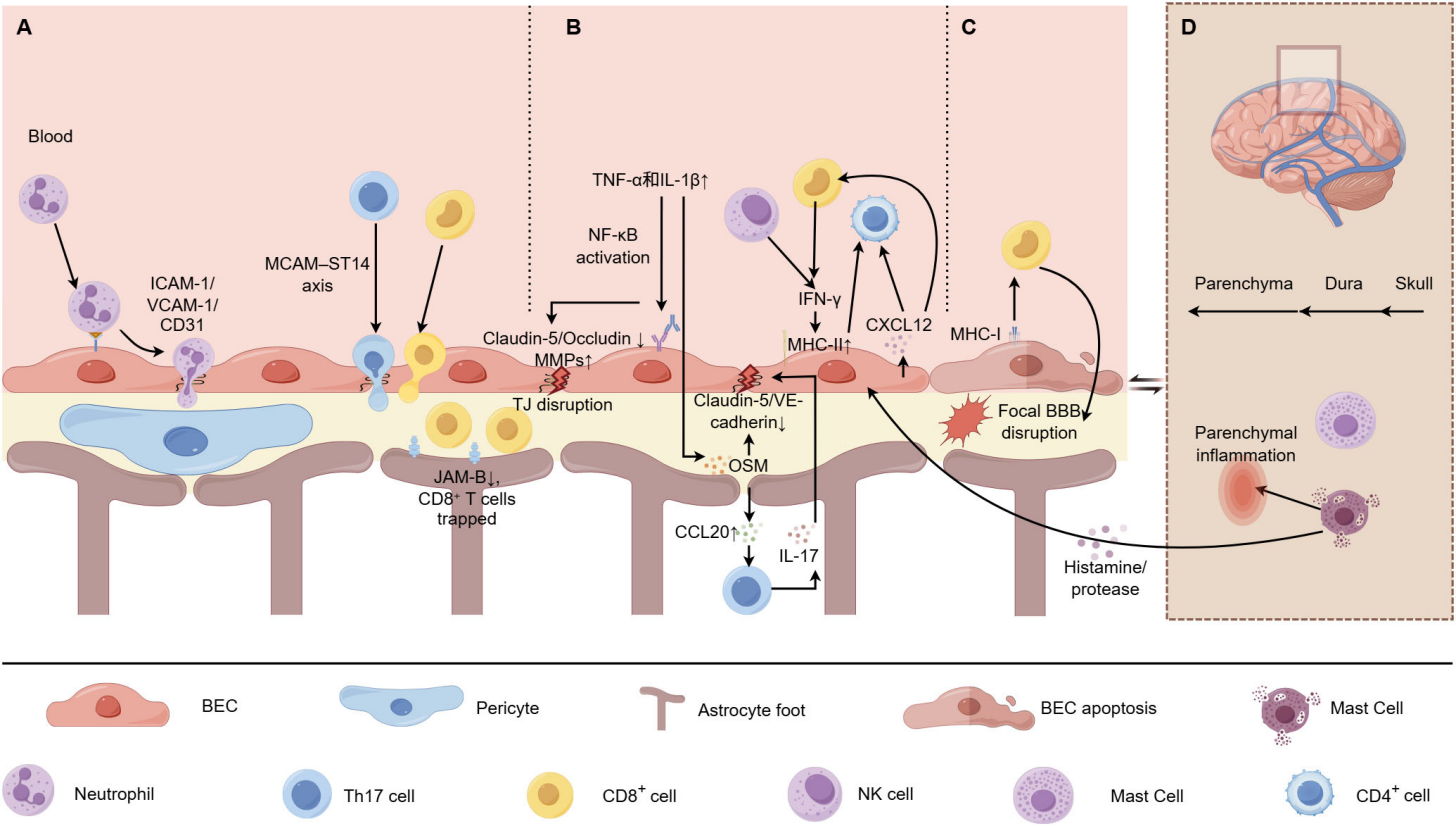
Key mechanisms of immune-endothelial crosstalk in neuroinflammation. **(A)** Adhesion cascades govern multi-step leukocyte migration across the BBB; **(B)** Cytokine/chemokine networks orchestrate barrier disruption and directed immune recruitment; **(C)** Endothelial antigen presentation directly activates T cells, triggering focal vascular injury; **(D)** Meningeal immune cells relay systemic signals via CSF dynamics and local mediator release. MMPs, Matrix Metalloproteinases; TJ, Tight Junction.

The initiation and progression of neuroinflammation are contingent upon a meticulously coordinated molecular dialogue between immune cells and brain endothelial cells (BECs). This interaction reconceptualizes the brain microvascular endothelium, traditionally perceived as a static barrier, into a dynamic regulatory interface for neuroimmune responses (1, 2). This section seeks to systematically elucidate the complex mechanisms underlying this dialogue, ranging from direct interactions at the vascular wall mediated by adhesion molecules, cytokines, and antigen presentation, to systemic influences exerted by specialized immune cells at the meningeal interface through the regulation of cerebrospinal fluid dynamics. Collectively, these mechanisms illuminate the fundamental regulatory network of the immune-endothelial axis in both physiological and pathological contexts (3, 4, 17) (**Figure 1**).

### 2.1 Adhesion molecule cascades: from classical pathways to emerging mechanisms

Leukocyte migration across the BBB is a complex, multi-step process that includes initial tethering, rolling, activation, firm adhesion, and subsequent transendothelial migration. Classical inflammatory cytokines, such as TNF-α and IL-1β, activate BECs, leading to the upregulation of traditional adhesion molecules like ICAM-1 and VCAM-1, which facilitate firm adhesion of immune cells (18, 19). Recent research has identified additional critical molecules, thereby significantly enhancing our comprehension of the mechanisms underlying immune cell infiltration. Among these classical molecules, platelet endothelial cell adhesion molecule-1 (PECAM-1, also known as CD31) plays a well-established role in regulating the final phase of transendothelial migration. Experimental models of ischemic brain injury have demonstrated that genetic knockout or antibody-mediated blockade of PECAM-1 specifically impedes neutrophil infiltration into the parenchyma without affecting their adhesion within the vasculature, underscoring its function as the “final gatekeeper” of migration (20). Further investigations suggest that CD31 is a crucial molecule in the BBB’s response to pathological stimuli, and its dysfunction may mediate neuroinflammation, synergistically increasing AD risk with genotypes such as the Apolipoprotein E ϵ4 allele (ApoE4) (21).

In addition to these findings, novel adhesion pathways continue to be identified. For instance, CLMP is upregulated on brain and meningeal endothelial cells under inflammatory conditions and promotes immune cell migration across the barrier—a process that can be functionally blocked by specific antibodies. In patients with multiple sclerosis, CLMP-expressing immune cells localize to perivascular and meningeal infiltrates, and circulating CLMP-positive B lymphocytes and monocytes are increased, underscoring a clinically relevant role for this adhesion molecule in neuroinflammatory disease (22).

Another critical molecule, the melanoma cell adhesion molecule (MCAM/CD146), is expressed on specific subsets of BECs in both experimental autoimmune encephalomyelitis (EAE) lesions and MS lesions (23). Research indicates that cerebral endothelial MCAM preferentially facilitates the transmigration of pathogenic Th17 cells across the BBB by interacting with its immune ligand, tumor suppressor 14 (ST14), a serine protease, on infiltrating CD4+ T cells. Targeting the MCAM–ST14 axis presents a novel strategy for selectively inhibiting the central nervous system infiltration of autoreactive T cells (24).

The regulation of adhesion is characterized by remarkable cellular and spatial specificity. For instance, junctional adhesion molecule-B (JAM-B) is predominantly expressed on astrocytes that form the glia limitans, rather than on the endothelial cells of the BBB themselves. Research indicates that a deficiency in JAM-B does not inhibit the initial arrest or extravasation of CD8+ T cells at the BBB. Instead, it restricts these cells to perivascular and subarachnoid spaces, thereby reducing parenchymal autoimmune attacks while partially maintaining immune surveillance at the border regions. This discovery provides valuable insights for the development of targeted therapies aimed at limiting neuroinflammation without inducing systemic immunosuppression (25) (**Figure 1A**).

### 2.2 Cytokine and chemokine networks: orchestrating the directed flow of inflammation

A complex network of cytokines and chemokines intricately orchestrates the interaction between immune cells and endothelial cells. This system not only disrupts BBB integrity directly but also facilitates the directional migration and positioning of immune cells by establishing chemical gradients, thus functioning as the central command for the directed “flow” of neuroinflammation (4, 5).

Pro-inflammatory cytokines, such as TNF-α and IL-1β, are significant contributors to the disruption of the BBB. These cytokines activate the NF-κB signaling pathway, triggering downstream transcriptional programs that upregulate pro-inflammatory mediators and matrix metalloproteinases (MMPs) (26). Among the consequences are the internalization and degradation of tight junction proteins, including claudin-5 and occludin, as well as the proteolytic activity of MMPs, particularly MMP-2 and MMP-9, which degrade the vascular basement membrane. Collectively, these effects compromise tight junction integrity and increase paracellular permeability, thereby facilitating the entry of immune cells and inflammatory mediators into the brain parenchyma—hallmarks of acute barrier disruption (27). In addition to such acute effects, early-life exposure to IL-1β may have enduring developmental implications. Research conducted by Fetsko et al., utilizing a zebrafish model, demonstrated that embryonic exposure to IL-1β impairs normal BBB development by inhibiting endothelial Wnt/β-catenin signaling. This finding establishes a direct mechanistic connection between early-life inflammation and an increased risk of neurological disorders in adulthood (28).

Furthermore, synergistic and positive feedback interactions exist among cytokines. For instance, IL-17 and TNF-α synergistically enhance endothelial permeability (29). Research by Hermans et al. identified a significant pathogenic positive feedback loop involving oncostatin M (OSM), an IL-6 family cytokine produced by activated myeloid cells and astrocytes. OSM downregulates claudin-5 and VE-cadherin while inducing BECs and astrocytes to secrete the Th17 cell chemokine CCL20. This creates a self-amplifying cycle: OSM disrupts the barrier and recruits Th17 cells, which produce IL-17, further exacerbating inflammation and barrier damage (30).

Interferon-gamma (IFN-γ), classified as a type II interferon, augments the antigen-presenting capabilities of BECs by upregulating major histocompatibility complex (MHC) class II molecules, thereby potentially facilitating interactions with CD4^+^ T cells (31). Simultaneously, accumulating evidence highlights the pivotal role of endothelial cells in mediating the pathological effects of type I interferon signaling within the brain (32). Research by Viengkhou et al. has demonstrated that the cerebral microvascular endothelium serves as a primary mediator of type I interferon-induced neurotoxicity. Specifically, brain-derived interferon-alpha directly induces microangiopathy via endothelial type I interferon receptor signaling, and the endothelial-specific ablation of this receptor mitigates vascular pathology and extends survival in murine models (33). Moreover, single-cell transcriptomic analysis conducted by Quan et al. in Parkinson’s disease models has identified robust type I interferon response signatures in both microglia and endothelial cells, thereby associating this pathway with a broader spectrum of neurodegenerative disorders (34).

The function of chemokines demonstrates significant dependency on the disease context. The C-X-C motif chemokine ligand 12 (CXCL12) and its receptor, C-X-C chemokine receptor type 4 (CXCR4), constitute a critical axis in the regulation of immune cell homing (35, 36). Research conducted by Li et al. indicates that post-subarachnoid hemorrhage, CXCL12 produced by brain microvascular endothelial cells and pericytes recruits CD8+ T cells, thereby exacerbating BBB dysfunction (36). In contrast, studies by Wang et al. in ischemic stroke models reveal that endothelial-derived CXCL12 plays a vital role in recruiting protective natural killer cells and type 3 innate lymphoid cells to the lesion site, thus enhancing outcomes (35). This duality highlights the imperative of meticulously considering the disease context when therapeutically targeting this pathway (**Figure 1B**).

### 2.3 Antigen presentation at the blood-brain barrier: from gatekeeper to activator

BECs are not solely structural barriers; they also actively engage in adaptive immune responses. By presenting antigens and directly interacting with T cells, BECs transition from passive “gatekeepers” to active “activators” of the immune response, significantly affecting the progression of neuroinflammation (7, 37). Pioneering research by Aydin et al. demonstrated that BECs are capable of cross-presenting exogenous antigens to CD8+ T cells via MHC class I molecules. Under conditions of physiological shear stress, this antigen-specific recognition results in the arrest of CD8+ T cell migration, their activation, and ultimately induces BECs apoptosis and local disruption of the BBB. This mechanism highlights a direct, antigen-driven mode of injury to the neurovascular unit, independent of the classical trans-migration pathway (37). Single-cell transcriptomic analyses provide comprehensive evidence for the dynamic and diverse roles of BECs in neuroinflammation. Within the EAE model, researchers have identified a subpopulation of venous endothelial cells characterized by a distinct pro-migratory transcriptional profile. This discovery elucidates the intricate network of ligand-receptor interactions between these endothelial cells and infiltrating immune cells, reinforcing the notion that BECs are highly active and adaptable participants in neuroimmune communication (11) (**Figure 1C**).

### 2.4 Meningeal interface cells: a systemic extension of immune-endothelial dialogue

Beyond the traditional framework of interactions at the cerebral vascular wall, specialized immune cell populations at the meningeal interface—specifically mast cells and meningeal macrophages—represent a critical anatomical and functional extension of the immune-endothelial axis (38, 39). These cells, located at the boundary between the CNS and the periphery, significantly influence the homeostasis and progression of diseases within the neurovascular unit by actively modulating cerebrospinal fluid (CSF) dynamics and initiating local immune responses (17, 40).

Mast cells, as pivotal sentinels at the meningeal interface, demonstrate a distinct duality and context-dependent functionality. In response to sterile injuries, such as post-operative conditions or ischemic stroke, these cells rapidly degranulate, releasing histamine, proteases, and pro-inflammatory mediators (e.g., IL-17A). These substances can directly interact with BECs, leading to the disruption of tight junctions and a significant increase in barrier permeability, thereby exacerbating the neuroinflammatory cascade (41, 42). Recent studies have notably identified that dural mast cells can remotely induce brain parenchymal inflammation through specific receptor signaling along the “dura-brain axis,” offering mechanistic insights into their role as a conduit between peripheral and central immune events (43). Conversely, in the presence of infectious threats such as bacterial meningitis, these activation pathways may adopt a protective role by redirecting CSF flow and swiftly recruiting neutrophils to restrict pathogen spread (17). This functional plasticity indicates that the interaction between mast cells and the endothelium is not static but undergoes dynamic reprogramming, highly influenced by microenvironmental signals (38)°.

Meningeal macrophages, in collaboration with mast cells, are essential for maintaining CSF-lymphatic drainage homeostasis. Various macrophage subsets located within the dura mater and pia mater work synergistically to ensure the efficient drainage of CSF into meningeal lymphatic vessels by clearing metabolic waste and modulating the local immune microenvironment (44). The functional integrity of this “immune-lymphatic-vascular” axis is vital for the clearance of potentially neurotoxic substances from the brain and for the regulation of perivascular inflammation levels (39, 40). Dysfunction within this axis can result in abnormal protein aggregation and the retention of inflammatory mediators, which indirectly but persistently exacerbate endothelial cell stress and barrier dysfunction, thereby creating a vicious cycle that contributes to chronic neurodegenerative pathology. Consequently, the meningeal interface should not be viewed merely as a physical extension of immune-endothelial interactions; rather, it serves as a functional platform that systematically influences CNS immune homeostasis by regulating fluid circulation and material exchange (**Figure 1D**).

## 3 Aging: remodeling the immune-endothelial axis via intrinsic stress and inflammatory microenvironment

**Figure 2:**
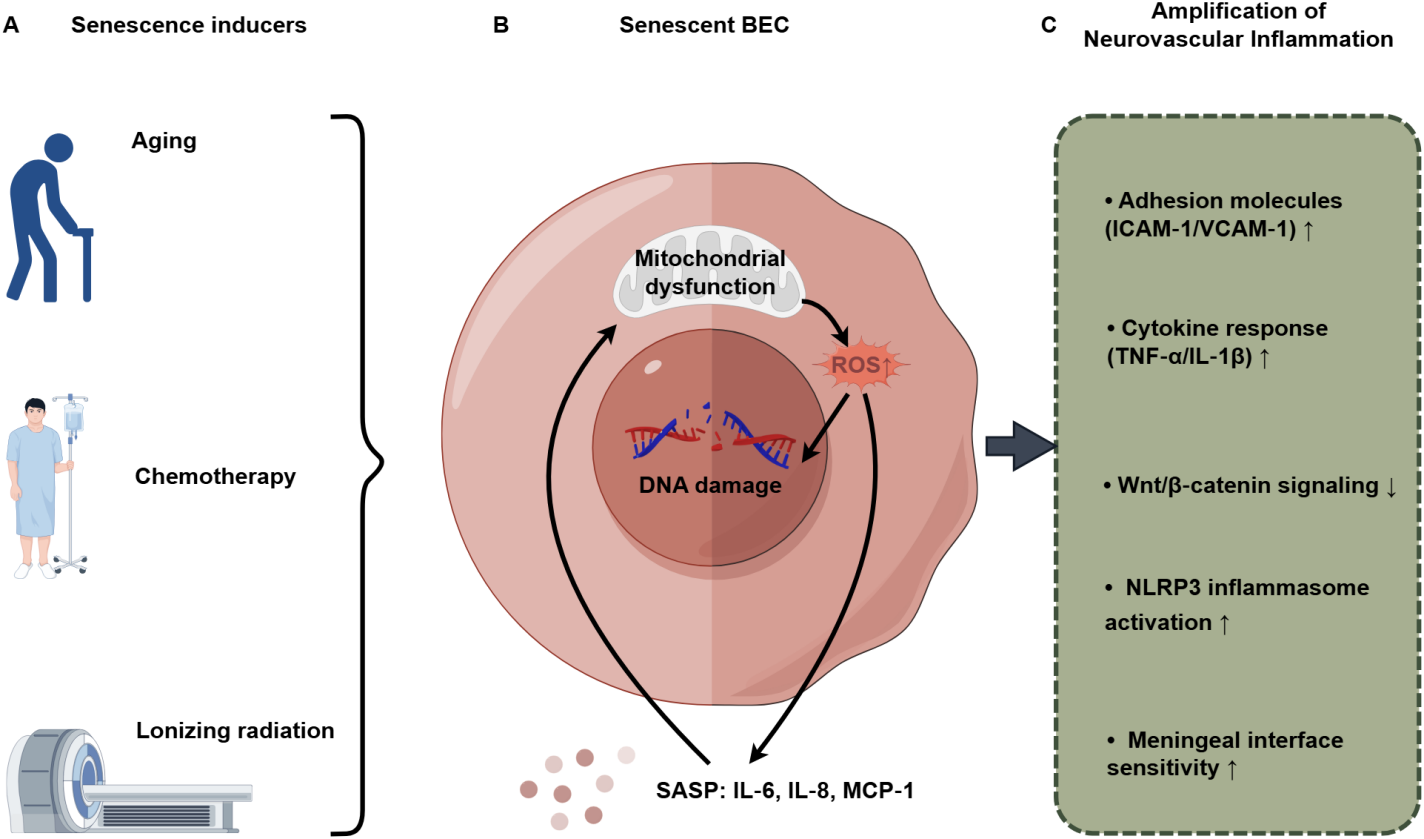
Endothelial senescence reprograms the immune−endothelial axis through a self−amplifying stress−inflammatory loop in the aging brain. **(A)** Systemic and environmental stressors initiate BEC senescence. **(B)** A self−reinforcing loop between mitochondrial dysfunction and SASP sustains a pro−senescent, pro−inflammatory niche. **(C)** This niche broadly amplifies immune−endothelial crosstalk, potentiating the neuroinflammatory pathways detailed in **Figure 1**.

Aging should not be regarded as a static background variable; rather, it is an active process that remodels the neurovascular unit. This dynamic process substantially reduces the threshold for neurovascular dysfunction and significantly amplifies the pathological effects of immune-endothelial interactions in both cerebrovascular and neurodegenerative diseases (45, 46). This section seeks to elucidate the fundamental role of aging in regulating the homeostasis and imbalance of the immune-endothelial axis. It does so by inducing mechanisms such as endothelial cell senescence and mitochondrial dysfunction, thereby establishing a systemic pro-inflammatory microenvironment (**Figure 2**).

### 3.1 Cellular senescence and the senescence-associated secretory phenotype: driving chronic low-grade inflammation

During the process of organismal aging, an increasing number of BECs enter a state of irreversible growth arrest known as cellular senescence, which is recognized as a fundamental driver of vascular aging (47). Importantly, senescent cells are not metabolically inactive; rather, they develop a pronounced pro-inflammatory secretory profile referred to as the senescence-associated secretory phenotype (SASP). The SASP is characterized by the abundant secretion of pro-inflammatory cytokines, such as IL-6, chemokines including IL-8 and monocyte chemoattractant protein-1 (MCP-1), as well as proteases (48). This secretory activity fosters a persistent, low-grade inflammatory microenvironment within the neurovascular unit, which continuously recruits and activates both peripheral and brain-resident immune cells. Consequently, a self-perpetuating vicious cycle is established: senescent endothelial cells promote inflammation, which in turn exacerbates endothelial dysfunction and vascular damage (49). Targeting cellular senescence, particularly through the inhibition of the SASP, has emerged as a promising strategy for mitigating age-related vascular inflammation. For example, research conducted by Matacchione et al. demonstrated that ginger extract exhibits “senomorphic” activity on human endothelial cells, significantly reducing the secretion of key SASP factors such as IL-8 and MCP-1. This finding suggests that dietary interventions may help alleviate the endothelial inflammatory burden associated with aging (50). It is important to recognize that cellular senescence is not solely induced by chronological aging but can also be accelerated by external stressors. The study by Ahire et al. provides direct evidence for this phenomenon, demonstrating that paclitaxel chemotherapy accelerates the senescence process in the mouse cerebral microvasculature. This accelerated cerebrovascular senescence was causally linked to impaired neurovascular coupling, BBB disruption, and cognitive dysfunction, thereby revealing a potential shared vascular pathological mechanism underlying “chemobrain” (51) (**Figure 2B**).

### 3.2 Mitochondrial dysfunction and oxidative stress: the intrinsic engine for immune activation

The decline in mitochondrial function within BECs as age progresses is a distinguishing characteristic of the aging endothelium (52). Dysfunctional mitochondria result in diminished efficiency of oxidative phosphorylation and an overproduction of ROS, which induces sustained oxidative stress. This intrinsic oxidative stress not only directly damages cellular macromolecules and oxidizes tight junction proteins, thereby compromising the integrity of the BBB, but also serves as an endogenous danger-associated molecular pattern (DAMP). This, in turn, activates innate immune pathways, including the NLRP3 inflammasome (53, 54). Mitochondrial dysfunction acts as an intrinsic link between aged endothelial cells and the hyperactivation of the innate immune system, thereby intensifying the inflammatory response within the neurovascular unit. This pathological process can be significantly exacerbated by external environmental factors (**Figure 2B**). For instance, Wang QQ et al. reviewed the mechanisms by which ionizing radiation induces senescence in brain cells, including endothelial cells, emphasizing that persistent oxidative stress, mitochondrial damage, and subsequent activation of the SASP are critical sequential events leading to neurovascular functional deficits and cognitive decline (55). This suggests that, irrespective of whether the senescence driver is intrinsic or extrinsic, mitochondrial damage and oxidative stress represent a common core nexus for amplifying downstream immune-endothelial inflammatory responses (**Figure 2C**).

The age-associated remodeling of the immune-endothelial axis—characterized by endothelial senescence, a persistent pro-inflammatory SASP microenvironment, and mitochondrial dysfunction—does not operate in isolation but actively primes the neurovascular unit for dysfunction across a spectrum of neurological disorders. As detailed in the following section, this age-related vulnerability manifests distinctly in acute conditions such as stroke, in chronic neurodegeneration such as AD, and in autoimmune conditions such as MS, where aging lowers the threshold for endothelial activation and amplifies the pathological consequences of immune-endothelial crosstalk.

## 4 The immune-endothelial axis in disease: from acute inflammation to autoimmune attack

Dysregulated interactions between the immune system and endothelial cells form a common pathological basis for various neurological diseases. However, the specific molecular mechanisms and cellular characteristics vary significantly across these disorders, resulting in distinct pathological trajectories (5). This chapter employs a disease-spectrum perspective to systematically elucidate the diverse roles of the immune-endothelial axis in three representative neuropathological conditions: acute inflammatory activation, as observed in stroke and TBI; chronic vascular dysregulation, as seen in AD and vascular cognitive impairment; and specific autoimmune attacks, exemplified by MS. Additionally, this section examines the regulatory role of meningeal interface immune cells, such as mast cells, in mediating these interactions across various diseases. This analysis aims to uncover how immune and endothelial cells either collaborate or conflict, collectively driving neuroinflammation and vascular dysfunction within distinct disease contexts (**Table 1**).

**Table 1:** Differential engagement of the immune–endothelial axis in neurological diseases.

Mechanism/disease	Acute (stroke/TBI)	Chronic (AD/vascular cognitive impairment)	Autoimmune (MS)
Dominant immune response	Neutrophil-driven burst; protective lymphocyte recruitment	Low-grade inflammation; impaired metabolic waste clearance	Antigen-specific T-cell attack; endothelial intrinsic dysfunction
Key molecular mediators	PECAM-1, CXCL12-CXCR4, IL-33, dural mast cells	C3a/C3aR, mCRP, Wnt/β-catenin, meningeal macrophages	MCAM–ST14, JAM-B, TNF-α–TRPV4, A20
Barrier outcome	Acute tight-junction disruption; secondary inflammatory injury	Progressive leakage; chronic cognitive decline	Focal breakdown; relapsing-remitting disability
Meningeal interface role	Remote pro-inflammatory signaling (dural mast cells)	Glymphatic dysfunction → Aβ accumulation	To be fully elucidated

AD, Alzheimer’s disease; ApoE4, apolipoprotein E ϵ4 allele; Aβ, β-amyloid; C3a, complement component 3a; C3aR, C3a receptor; CCL20, C-C motif chemokine ligand 20; CNS, central nervous system; CSF, cerebrospinal fluid; CXCL12, C-X-C motif chemokine ligand 12; CXCR4, C-X-C chemokine receptor type 4; IL-33, interleukin-33; JAM-B, junctional adhesion molecule-B; MCAM/CD146, melanoma cell adhesion molecule; mCRP, monomeric C-reactive protein; MS, multiple sclerosis; PECAM-1/CD31, platelet endothelial cell adhesion molecule-1; ST14, tumor suppressor 14; TBI, traumatic brain injury; TNF-α, tumor necrosis factor-α; TRPV4, transient receptor potential vanilloid 4.

### 4.1 Acute inflammatory activation: the storm-like response in stroke and TBI

In the context of acute CNS injuries, such as stroke and TBI, the immune-endothelial axis is rapidly and intensely activated, initiating a “storm-like” inflammatory response. This response is intended to clear damaged tissue but is often accompanied by secondary injury. Within this framework, BECs serve a dual function: they act as “sensors” of tissue damage and as “gateways” for the influx of immune cells into the parenchyma (56). Beyond the classic immune cell responses involving neutrophils and macrophages, meningeal mast cells located at the dural interface perform unique regulatory roles in acute neuroinflammation. Research suggests that following ischemic stroke, dural mast cells can detect injury signals through specific receptors and subsequently transmit pro-inflammatory signals along the “dura-brain axis,” thereby exacerbating parenchymal inflammation and BBB dysfunction from a distance (43). This mechanism highlights the functional crosstalk between the meningeal interface and the brain parenchyma in acute neuroinflammation, thereby broadening the spatial scope of the immune-endothelial axis in the pathology of stroke.

Neutrophils, as the initial responders within the innate immune system, engage in critical interactions with the endothelium. They contribute to early tissue damage by obstructing microvessels, releasing ROS, and forming neutrophil extracellular traps (NETs). However, they may also play a role in later tissue repair processes, highlighting their complex functional duality (57, 58). Key molecular mechanisms that regulate neutrophil infiltration, such as the “gatekeeper” function of PECAM-1 (CD31) in transendothelial migration, have been identified as promising therapeutic targets. Research indicates that inhibiting PECAM-1 following a stroke specifically reduces neutrophil infiltration into the brain without affecting their adhesion, thereby effectively alleviating ischemic brain injury (20).

The immune landscape of acute injury is more intricate than previously understood, involving complex interactions between endothelial cells and various immune cell subsets (59). For example, CXCL12 derived from BEC, through its receptor CXCR4, can actively recruit protective natural killer (NK) cells and innate lymphoid cell subsets to the ischemic site, illustrating the beneficial regulation by the immune-endothelial axis in mitigating damage (35). Simultaneously, macrophage subsets, particularly those derived from monocytes, serve as crucial mediators in preserving the integrity of the BBB and curbing the early expansion of infarcts, primarily through interleukin-33 signaling pathways. The recruitment of these macrophages is contingent upon the activation state of endothelial cells (60, 61). This suggests that, amidst the disorder of acute inflammation, there exists an “ordered” dimension to immune-endothelial interactions that facilitates repair processes.

In cases of hemorrhagic CNS injuries, the immune-endothelial axis is similarly robustly activated, although its specific mechanisms of action differ depending on the type of injury (36, 62, 63). For instance, in hemorrhagic stroke models, specialized vascular interfaces such as the ependyma have been identified as pivotal in initiating early inflammatory responses, with their activation occurring prior to the formation of perilesional edema (62). Moreover, in cerebral vascular malformations such as cavernous angiomas, studies have identified neuroinflammation as a fundamental driver, wherein interactions between inflammatory astrocytes and lesional endothelium can precipitate subsequent leukocyte recruitment and immunothrombosis (63). In models of subarachnoid hemorrhage, single-cell sequencing analyses have revealed that CXCL12, derived from intracranial microvasculature, directly facilitates the infiltration of CD8+ T cells and exacerbates BBB dysfunction (36). These findings collectively suggest that, despite the heterogeneity observed among various hemorrhagic injuries, targeting specific interaction nodes between immune cells and the endothelium may offer promising strategies for mitigating secondary neural damage.

Following TBI, the inflammatory response, while sharing certain similarities with ischemic stroke, demonstrates distinct spatiotemporal characteristics (64). The adhesive interactions between neutrophils and endothelial cells, mediated via the β2 integrin-ICAM-1 axis, play a critical role in the progression of TBI pathology. Inhibiting this specific interaction not only aids in maintaining BBB integrity and reducing ROS but also prevents the formation of NETs at their source, thereby providing a precise molecular target for addressing post-traumatic neuroinflammation (65).

### 4.2 Chronic vascular dysregulation: the insidious disruption in ad and vascular cognitive impairment

In contrast to the acute “storm,” the dysregulation of the immune-endothelial axis in AD and vascular cognitive impairment is characterized by a low-grade, chronic process that persists over several decades. This “insidious disruption” arises from a gradual, persistent abnormal interaction between the peripheral system and the CNS, which progressively undermines the homeostasis of the neurovascular unit and ultimately results in cognitive decline (66, 67). Beyond BBB dysfunction, emerging evidence points to the glymphatic system—a perivascular fluid transport network that clears metabolic waste from the brain—as a key player in chronic neurodegeneration (68). This system operates in close coordination with meningeal lymphatic vessels, which drain solutes from the cerebrospinal fluid to peripheral lymph nodes (39, 44). When glymphatic clearance is impaired, toxic proteins such as β-amyloid accumulate in perivascular spaces, creating a pro-inflammatory milieu that places sustained stress on the endothelium and exacerbates neurovascular dysfunction (39, 68). Aging is a major factor that compromises glymphatic efficiency, and genetic susceptibility, particularly the APOE4 variant, further impairs perivascular drainage and meningeal lymphatic function (68). Together, these observations delineate an “immune-lymphatic-vascular” axis in which dysfunction at any level perpetuates chronic inflammation and contributes to the pathogenesis of age-related neurodegenerative diseases.

The complement system, an essential component of innate immunity, plays a pivotal role in chronic pathological conditions (69). Notably, age-associated dysregulation of complement component C3a and its receptor C3aR signaling can lead to increased expression of VCAM-1 by endothelial cells, facilitate lymphocyte infiltration, and disrupt vascular endothelial cadherin junctions. These processes collectively compromise the integrity of the BBB, thereby fostering an environment conducive to neurodegenerative alterations (70). Additionally, monomeric C-reactive protein (mCRP) emerges as a significant peripheral mediator, accumulating in the brain during neuroinflammatory states. Its presence is strongly correlated with BBB disruption and exacerbated β-amyloid pathology, including the formation of mixed Aβ-mCRP plaques (71, 72). Consequently, mCRP serves as both a biomarker and a potential molecular link connecting systemic inflammation, BBB dysfunction, and AD pathology.

From the perspective of intrinsic endothelial signaling pathways, the Wnt/β-catenin pathway, which is essential for maintaining BBB homeostasis, is frequently dysregulated in AD (13, 14). This dysregulation may be associated with functional abnormalities in specific endothelial proteins. For instance, PECAM-1 (CD31) is regarded as a regulatory protein responsive to changes in the BBB. Under the influence of the ApoE4 genetic background and systemic inflammation, dysregulation of its signaling may predispose the neurovascular unit to a pro-inflammatory and permeable state, thereby elevating disease risk (21). Genetic studies offer more direct evidence: the P460L variant of the AD risk-associated gene EPHA1 compromises the function of its protein product in regulating T-cell recruitment and maintaining barrier integrity through reverse signaling, thus directly linking a genetic risk locus to functional deficits in the immune-endothelial axis (73). Collectively, these mechanisms illustrate a pathological scenario characterized by chronic immune activation, dysregulated endothelial signaling, and the gradual deterioration of barrier function.

### 4.3 Specific autoimmune attack: targeted breach in MS

MS exemplifies an extreme paradigm of immune-endothelial interaction, characterized by a targeted assault on the CNS initiated by autoreactive T cells. In this context, the BBB endothelial cells are not merely passive entities; rather, they actively contribute to the onset of the disease. Their functional state significantly influences the magnitude and extent of the autoimmune response (74, 75). Recent research has identified that a subset of BECs adopts a distinct pro-migratory phenotype during the disease process. For example, certain endothelial cell subsets expressing the melanoma cell adhesion molecule specifically recruit pathogenic CD4+ T lymphocytes through interaction with their ligand ST14, functioning as a “Trojan gate” that facilitates the entry of autoreactive cells into the CNS. Single-cell transcriptomic analyses further elucidate the functional heterogeneity among endothelial cells in the context of neuroinflammation, with venous endothelial cells exhibiting particularly notable gene expression changes related to antigen presentation and interferon response. This suggests that different vascular segments play unique roles in orchestrating immune attacks (11).

A notable advancement in the understanding of MS pathogenesis is the identification of intrinsic defects in endothelial cells derived from MS patients. Research indicates that endothelial-like cells generated from MS patient-derived hiPSCs inherently exhibit compromised junctional integrity, diminished barrier function, and pro-inflammatory characteristics, even in the absence of immune cell-mediated attack. These defects can be partially ameliorated through the activation of the Wnt/β-catenin signaling pathway (13, 76, 77). This suggests that an inherently “susceptible” or “dysfunctional” state of BECs is a fundamental aspect of MS pathogenesis.

The complexity of MS is exemplified by intricate, multi-layered, and multi-cellular regulatory mechanisms. Microglia-derived TNF-α can enhance the expression of transient receptor potential vanilloid type 4 channels on endothelial cells, thereby exacerbating the disruption of the BBB (78). The endothelial ubiquitin-editing enzyme A20 functions as an intrinsic regulatory mechanism, mitigating excessive T cell adhesion by restricting the expression of co-stimulatory molecule ligands (79). Additionally, the precise regulation of CD8+ T cell migration by JAM-B at the glia limitans illustrates the complex defense system of CNS barriers and provides therapeutic insights for controlling parenchymal inflammation while maintaining essential immune surveillance (25). Collectively, these findings elucidate that in MS, the interaction between the immune system and endothelial cells constitutes a highly specific “battle of attack and defense,” occurring at distinct cellular subsets and molecular nodes.

## 5 Diagnostic innovations: multi-dimensional assessment of the immune-vascular axis

A precise evaluation of immune-endothelial interactions is essential for comprehending the pathological progression of neuroinflammation and vascular dysfunction, as well as for advancing precision medicine. The conventional diagnostic paradigm, which relies solely on the concept of a “leaky barrier,” is no longer adequate. Contemporary diagnostics are transitioning towards a multi-dimensional and dynamic approach that incorporates three technological pillars: liquid biopsy, advanced neuroimaging, and human-relevant *in vitro* models. These tools collectively facilitate a comprehensive understanding of the complex interplay within the immune-vascular axis at molecular, cellular, and systemic levels (80). This integrated assessment framework is critically important for early disease detection, accurate patient stratification, and dynamic monitoring of therapeutic efficacy.

### 5.1 Liquid biopsy: capturing systemic and cns-specific biological signals

Liquid biopsy technologies, through the analysis of biomarkers present in peripheral blood and CSF, provide a minimally invasive approach to evaluating the immune-endothelial axis both systemically and within the CNS. Biomarkers in peripheral blood offer a practical method for assessing systemic endothelial activation and immune status. Circulating levels of soluble adhesion molecules, such as soluble ICAM-1 and soluble VCAM-1, act as indirect indicators of endothelial activation (2). More specific markers, such as mCRP, which deposits in cerebral vessel walls and directly correlates with neurodegenerative disease activity, provide insights beyond those offered by traditional CRP (72). Furthermore, the detection of endothelial-derived extracellular vesicles shows potential for more accurately reflecting the physiological and pathological state of the endothelium *in vivo* (81).

CSF proteomic and metabolomic analyses offer a more precise insight into intrathecal pathology. CSF exchanges directly with brain interstitial fluid, and alterations in its composition can more accurately reflect localized events within the neurovascular unit. For example, machine learning analysis of extensive CSF proteomic datasets can identify distinct protein signatures in individuals at elevated risk for AD, such as carriers of the APOE4 allele. These signatures are enriched with proteins derived from endothelial cells, glial cells, and peripheral immune cells, and they are independent of traditional amyloid or tau pathology. This independence suggests that these signatures may represent an early vulnerable state characterized by combined neurovascular and immune dysregulation (82).

Comprehensive phenotyping of peripheral immune cells may act as a “peripheral mirror” reflecting the immunopathological state of the CNS. Variations in the quantity and functionality of specific immune cell subsets frequently correlate with the progression of CNS diseases. For instance, a decrease in circulating non-classical monocytes is significantly associated with the severity of HIV-related cerebral small vessel disease, illustrating the clinical utility of detailed immunophenotyping in evaluating overall immune status and its effects on the endothelium (83). In MS, peripheral frequencies of Th1 and Th17 CD4^+^ T cells have been proposed as biomarkers of disease activity, with higher frequencies correlating with clinical relapse rates and radiographic disease burden (84, 85). In AD, altered proportions of non-classical monocytes and reduced regulatory T cell frequencies have been associated with accelerated cognitive decline and increased brain amyloid burden (86, 87). The translational relevance of these peripheral phenotypes lies in their interface with the endothelium: activated T cells expressing specific chemokine receptors are recruited to the CNS via cognate chemokines upregulated on activated brain endothelial cells, directly linking systemic immune profiles to localized neuroinflammation.

### 5.2 Advanced neuroimaging: from structural visualization to functional and molecular imaging

Recent advancements in neuroimaging are significantly enhancing the evaluation of the BBB and neuroinflammation, transitioning from macroscopic structural observations to the quantitative characterization of microscopic functions and molecular pathologies. Techniques for assessing the integrity of the barrier are continually evolving. Dynamic contrast-enhanced magnetic resonance imaging (MRI) remains the clinical gold standard for quantifying BBB permeability and has demonstrated early barrier disruption in conditions such as mild cognitive impairment and AD (5). Emerging methodologies, including vessel-encoded arterial spin labeling and water-exchange MRI, are poised to offer more specific parameters for assessing BBB water exchange without the need for contrast agents, thereby providing novel tools for non-invasive evaluation of barrier function (88). Importantly, developments in imaging are expanding beyond the BBB to potentially assess the meningeal interface. For instance, the development of novel tracers that specifically target meningeal mast cell activation or quantitatively evaluate meningeal lymphatic drainage function could offer new insights into the functional state of the immune-endothelial axis at the broader meningeal-vascular interface (38, 40).

Molecular probes designed for imaging immune cell activity are crucial for the investigation of neuroinflammation. The 18-kilodalton translocator protein positron emission tomography imaging is extensively employed for visualizing neuroinflammation. Nevertheless, the origin of its signal is complex due to its constitutive expression in various cell types, including cerebrovascular endothelium, which necessitates careful interpretation of the results (89, 90). This complexity has prompted the development of novel tracers with enhanced cellular specificity. These include probes targeting the purinergic receptor P2X7 or the colony-stimulating factor 1 receptor, which aim to more accurately discern the activation states of specific immune cell populations, such as microglia and macrophages (91, 92).

The evolution of paradigms for interpreting imaging data is of paramount importance. Contemporary perspectives suggest transcending the binary “open” or “closed” BBB model, highlighting the necessity of comprehending barrier alterations through multiple dimensions, such as temporal dynamics, spatial heterogeneity, and mechanistic specificity. For example, transient remodeling of tight junctions induced by cytokines is pathologically distinct from the physical disruption caused by direct T-cell cytotoxicity. Future imaging technologies must possess the ability to discern such nuances to inform more targeted therapeutic strategies (37, 93).

### 5.3 Integrative *in vitro* models: platforms toward personalized assessment

*In vitro* models utilizing hiPSCs and bioengineered platforms represent transformative tools for investigating immune-endothelial interactions in a personalized context. Patient-specific hiPSC-derived BBB models are capable of recapitulating endothelial phenotypes reflective of individual genetic backgrounds. Models developed from cells of patients with MS or amyotrophic lateral sclerosis (ALS) have successfully reproduced inherent barrier defects, increased permeability, and pro-inflammatory characteristics, thereby elucidating dysregulation in critical signaling pathways such as Wnt/β-catenin (13, 14). These models facilitate *in vitro* diagnosis of barrier pathologies and enable personalized drug screening, effectively establishing a “patient-in-a-dish” model of the neurovascular unit (94).

**Figure 3:**
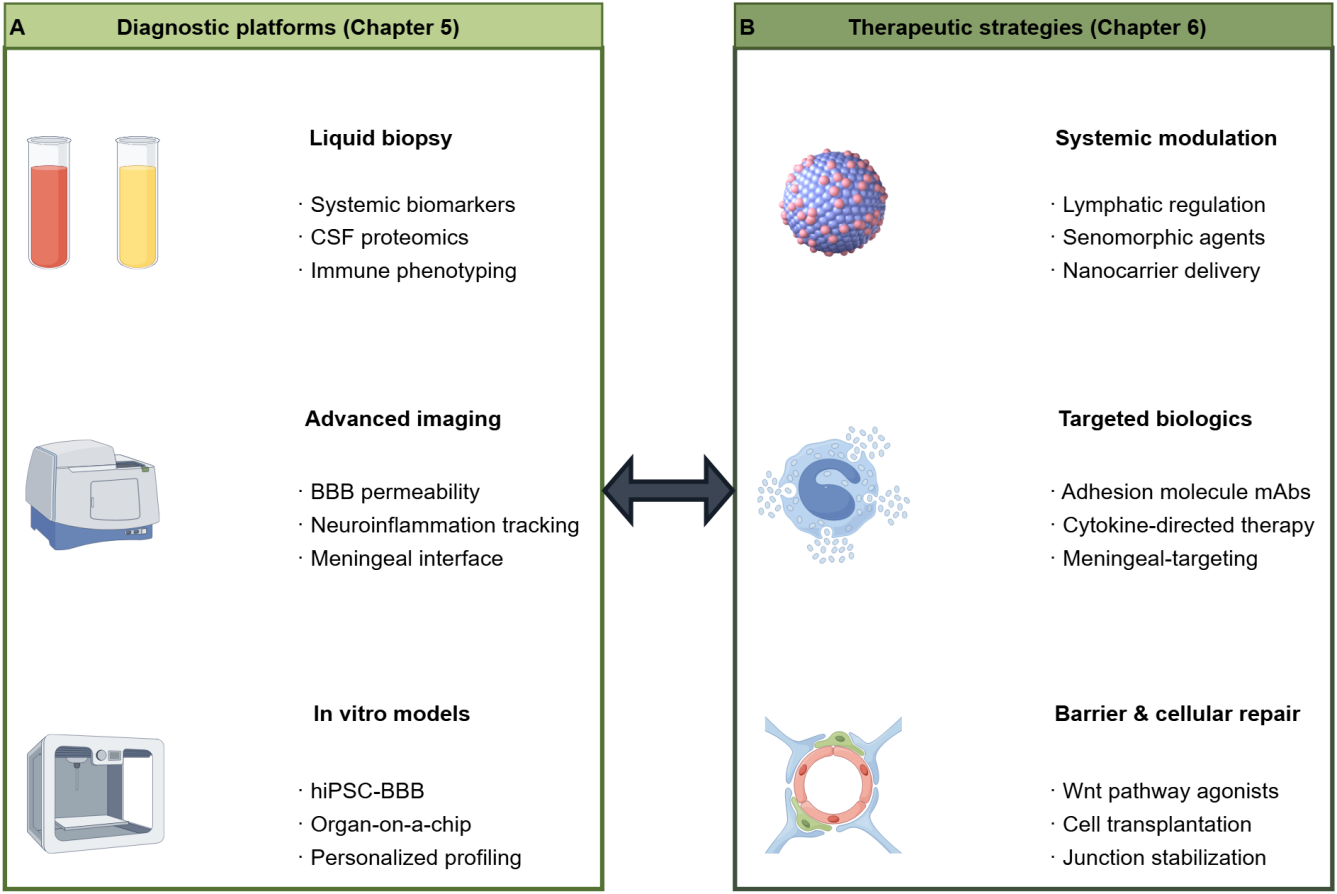
Integrated diagnostic−therapeutic framework for targeting the immune–vascular axis in neurological diseases. **(A)** Multidimensional diagnostic platforms span systemic biofluid analysis, neuroimaging, and human−relevant modeling. **(B)** Precision therapeutic strategies operate at systemic, targeted, and cellular/pathway levels.

Organ-on-a-chip and microfluidic technologies facilitate the construction of more physiologically relevant three-dimensional BBB models by incorporating multiple human cell types, such as endothelial cells, pericytes, and astrocytes, and by simulating physiological fluid shear stress and cell-cell interactions (95). These platforms allow for real-time, dynamic monitoring of immune cell migration, changes in barrier permeability, and intercellular communication. As such, they serve as powerful tools that bridge the gap between traditional cell culture and animal experiments, enabling high-throughput drug testing and mechanistic analysis (96, 97). These technologies are particularly well-suited for elucidating the specific contributions of different cell types within the neurovascular unit to the overall immune response (**Figure 3A**).

Over the past decade, organ-on-a-chip and microfluidic technologies have advanced considerably, enabling the construction of more physiologically relevant three-dimensional BBB models. These systems typically incorporate multiple human cell types—endothelial cells, pericytes, and astrocytes—cultured under physiological fluid shear stress, a critical factor for maintaining barrier phenotype and function (98). BBB-on-chip platforms enable real-time, dynamic monitoring of immune cell migration, barrier permeability via integrated electrodes or fluorescence imaging, and intercellular communication under controlled conditions. Complementing these approaches, hiPSC-derived brain organoids have been increasingly vascularized through co-differentiation or endothelial cell incorporation, generating vascularized organoid models that offer opportunities to study human-specific immune-endothelial interactions within a complex tissue context (99, 100). Each model system carries distinct strengths and limitations: 2D monolayers enable high-throughput screening but lack three-dimensional architecture and shear stress; organoids provide tissue complexity but face challenges in vascularization and reproducibility; microfluidic chips offer physiological flow and multi-cell co-culture but require specialized equipment. Collectively, these platforms serve as complementary tools that bridge the gap between traditional cell culture and animal models, enabling mechanistic dissection of immune-endothelial crosstalk and drug testing in human-relevant contexts (95, 97).

The model systems discussed above derive from diverse experimental approaches, each with inherent limitations that warrant consideration when extrapolating findings to human immune-endothelial interactions (2, 101). Murine models exhibit important species differences in BBB molecular composition, including expression levels of drug transporters and tight junction protein isoforms (102). Moreover, the mouse immune system differs from the human system in the relative abundance of lymphocyte subsets and the functional properties of innate immune cells (103). Zebrafish embryo studies offer advantages for high-throughput screening and optical clarity but lack the complexity of the mature mammalian neurovascular unit and are limited to developmental stages (104). hiPSC-derived models and organ-on-chip platforms address some of these limitations by incorporating human cells and physiological conditions, yet they remain reductionist relative to the intact organism and lack systemic immune components (105). These limitations underscore the necessity of integrating findings across complementary model systems and validating key mechanisms in human tissue samples or clinical cohorts before drawing conclusions about human pathophysiology.

Despite the promise of these multi-dimensional diagnostic approaches, several practical challenges must be addressed before they can be integrated into routine clinical practice. Biomarker specificity remains a major hurdle: soluble adhesion molecules such as sICAM-1 and sVCAM-1 are elevated across a wide range of inflammatory and cardiovascular conditions, limiting their utility as disease-specific markers of immune-endothelial activation. Emerging approaches using combinatorial biomarker panels or cell-type-specific extracellular vesicles may enhance specificity but require further validation (106, 107). Reproducibility across laboratories represents another critical challenge, particularly for imaging biomarkers such as dynamic contrast-enhanced-MRI-derived K-trans, which is influenced by scanner type, acquisition protocol, and analysis pipeline (108). Standardization initiatives, including consensus acquisition protocols and public imaging databases, are essential to enable multi-center studies and clinical adoption. Barriers to clinical implementation include the invasive nature of CSF collection, which limits its use to research settings or highly selected patient populations; the high cost and limited availability of advanced imaging techniques; and the lack of regulatory approval for most emerging biomarkers (109, 110). Addressing these translational barriers will require coordinated efforts among basic scientists, clinical researchers, regulatory agencies, and industry partners.

## 6 Therapeutic frontiers: reprogramming immune-vascular dialogue

Drawing on a comprehensive understanding of the intricate regulatory mechanisms that govern the immune-endothelial axis, therapeutic strategies addressing neuroinflammation and vascular dysfunction are experiencing a paradigm shift. As discussed in Section 5, the translation of preclinical findings to human therapies must account for species-specific differences and the inherent limitations of model systems. With this caveat in mind, emerging therapeutic concepts focus on stabilizing the BBB, reprogramming immune cell trafficking, and mitigating systemic vulnerabilities such as aging. The overarching objective is not the indiscriminate suppression of immune function but rather the restoration of nuanced immune homeostasis within the neurovascular unit (111). This chapter systematically delineates novel, multi-layered strategies, ranging from the targeting of specific molecules to the implementation of integrated systemic interventions. Beyond these direct intervention strategies, emerging principles for optimizing treatment timing based on circadian biology are also discussed as a future-oriented perspective.

### 6.1 Precision modulation of immune cell trafficking

Targeted intervention in the specific stages of immune cell transmigration across the BBB represents an optimal approach for reducing pathological infiltration while maintaining physiological immune surveillance. This strategy primarily relies on the development of biologics that target key adhesion molecules or signaling pathways. Monoclonal antibodies are central to this field. Although the anti-α4 integrin monoclonal antibody, natalizumab, has demonstrated efficacy in the treatment of MS, its association with an increased risk of progressive multifocal leukoencephalopathy has prompted the search for safer therapeutic targets (112). Antibodies targeting MCAM and JAM-B have shown considerable promise in preclinical studies. The former selectively inhibits the infiltration of pathogenic Th17 cells, whereas the latter restricts CD8+ T cells to the CNS border regions, thereby reducing parenchymal inflammation without completely obstructing immune surveillance (24, 25). These strategies aim to achieve “precision traffic control” by preventing the “unauthorized entry” of pathogenic immune cells.

Targeting critical cytokine pathways constitutes a significant avenue of research. For example, in the context of antibody-mediated autoimmune encephalitis, such as anti-N-methyl-D-aspartate receptor encephalitis, IL-1β serves as a principal mediator that contributes to the disruption of the BBB. Empirical evidence indicates that the administration of an IL-1 receptor antagonist, such as anakinra, can substantially mitigate barrier damage and alleviate neuropsychiatric symptoms in relevant models. This underscores the therapeutic potential of targeting pivotal inflammatory signals upstream of endothelial cells in specific pathological conditions (113).

### 6.2 Barrier reinforcement and homeostasis restoration strategies

Enhancing the intrinsic defense mechanisms of the BBB or repairing its damage constitutes a fundamental strategy for stabilizing immune-vascular interactions at the structural level. Activation of endogenous protective pathways represents a particularly promising approach. The Wnt/β-catenin signaling pathway plays a critical role in the development and maintenance of the BBB, with its reduced functionality observed in various disease models, including MS and ALS. Employing agonists of this pathway has been shown to effectively ameliorate barrier function defects in patient-derived cellular models, indicating its potential as a broad-spectrum disease-modifying therapy, particularly for conditions characterized by intrinsic barrier dysfunction (13, 14).

Targeting the direct mediators of barrier disruption has proven effective. For instance, the inhibition of non-muscle myosin light chain kinase can prevent the degradation of the tight junction protein claudin-5 in EAE, thereby stabilizing the BBB and reducing neutrophil infiltration (114). Recent research has revealed that certain non-classical molecules, such as the psychedelic compound N,N-dimethyltryptamine, exhibit neuroprotective effects through interaction with the Sigma-1 receptor. These effects are mediated by mechanisms such as the restoration of tight junction integrity and the reduction of neuroinflammation, thereby uncovering a novel therapeutic pathway for stroke treatment (115).

Addressing aging-associated endothelial dysfunction presents new opportunities for the management of age-related diseases. Senomorphics, which are compounds that inhibit the deleterious secretory phenotype of senescent cells without inducing cell death, have emerged as a promising therapeutic strategy supported by both *in vitro* and *in vivo* studies (116, 117). For instance, ginger extract has been shown to decrease the release of SASP factors from endothelial cells, providing a proof-of-concept for disrupting the “inflammation-senescence” cycle in the vasculature through dietary or pharmacological interventions (50). This strategy seeks to ameliorate the aged microenvironment at its origin, thereby enhancing the stress resistance and anti-inflammatory capacity of endothelial cells.

### 6.3 Frontier integrated and systemic intervention approaches

Advancements in therapeutic strategies have progressed beyond targeting individual molecules, with next-generation treatments encompassing innovative drug design, cell therapies, and the modulation of systemic homeostasis. Novel smart biologics epitomize the principle of precision delivery. For example, a bifunctional anti-VCAM-1/CD39 compound is engineered to specifically target the anti-inflammatory and antithrombotic ectonucleotidase CD39 to activated vascular endothelium. In models of cerebral ischemia, this approach effectively reduced inflammation, thrombus formation, and preserved the integrity of the blood-brain barrier by locally generating adenosine at the site of injury, thereby demonstrating the efficacy of smart design in achieving pharmacological effects “at the right time and place” (118).

Cell-based regenerative and immunomodulatory therapies present novel opportunities for tissue repair. The transplantation of hiPSC-induced vascular endothelial cells not only restores vascular architecture but also recruits regulatory T cells to ischemic white matter regions, thereby establishing an anti-inflammatory microenvironment that facilitates remyelination and functional recovery (119). This underscores the pivotal role of the intrinsic immunomodulatory capacity of healthy endothelial cells in the repair process.

Modulating meningeal interface immunity and fluid homeostasis offers a novel systemic perspective for therapeutic intervention. One approach involves targeting immune cells located within the dura mater, such as mast cells. For instance, the application of mast cell stabilizers, like disodium cromoglicate, has demonstrated potential in preclinical models to mitigate BBB disruption and enhance cognitive outcomes by inhibiting pathological activation of these cells (41, 120). Conversely, promoting the growth and functionality of meningeal lymphatic vessels, for example through vascular endothelial growth factor-C, can facilitate the clearance of toxic substances and inflammatory mediators from the brain. In stroke models, this strategy has been shown to effectively reduce brain edema, modulate neuroinflammation, and improve outcomes, suggesting that optimizing the brain’s “waste clearance system” can indirectly enhance immune-endothelial interactions (121).

Nanotechnology is progressively enhancing synergistic therapeutic strategies aimed at the neurovascular unit. Through the development of multifunctional nanodelivery systems, researchers are able to modulate immune-endothelial interactions at various levels. This is achieved by improving BBB penetration through the optimization of carrier interactions with brain endothelial cells, and by precisely delivering therapeutic agents to neuroimmune cells via targeting ligands. Such a design effectively addresses the dual challenges of traversing the endothelial barrier and navigating the complexities of the neuroinflammatory network, thereby offering a novel approach for the simultaneous modulation of multiple aspects of immune-endothelial communication (122) (**Figure 3B**).

### 6.4 Circadian regulation of the immune-endothelial axis: a chronotherapeutic perspective

Beyond the direct therapeutic strategies discussed above, the circadian clock has emerged as an important modulator of immune-endothelial interactions, with potential implications for optimizing treatment timing. Endothelial cells express core clock genes (e.g., BMAL1, CLOCK, PER, CRY), and BBB integrity exhibits diurnal variation, with tight junction protein expression and paracellular permeability fluctuating over the 24-hour cycle (123, 124). Immune cell trafficking also follows circadian patterns: the expression of adhesion molecules on endothelial cells and the availability of circulating leukocytes are both under clock control, resulting in time-of-day-dependent differences in leukocyte recruitment to inflammatory sites (125). These observations raise the possibility of chronotherapeutic strategies—the optimization of drug administration timing to align with circadian rhythms—to enhance efficacy and reduce toxicity of interventions targeting the immune-endothelial axis. For example, administration of endothelial-stabilizing agents during periods of maximal barrier permeability or immunomodulatory drugs at times of peak immune cell trafficking could theoretically improve therapeutic outcomes (126). While chronotherapy has been explored in cardiovascular disease and cancer, its application to neuroinflammatory and neurovascular disorders remains largely unexplored (127). Future studies integrating circadian biology with immune-endothelial research may open new avenues for optimizing existing therapies and developing novel treatment protocols.

## 7 Conclusion and future directions

This review identifies the immune-endothelial interface as a central regulator in neuroinflammation and vascular pathology, moving beyond its conventional role as a passive barrier to function as an active signaling platform that orchestrates neuroimmune communication. Our analysis elucidates how this dynamic interface integrates intricate molecular interactions—including emerging adhesion systems, spatially organized cytokine networks, and antigen presentation mechanisms—to regulate BBB integrity and immune cell trafficking in neurological disorders. The field is experiencing a rapid translation of these mechanistic insights, facilitated by advanced neuroimaging techniques that allow real-time visualization of endothelial activation states and targeted therapies that achieve precise immunomodulation in experimental models. These advancements highlight a paradigm shift from a barrier-centric perspective to recognizing the neurovascular unit as a complex hub of immune regulation.

To advance this promising field, future research should concentrate on several critical directions: (1) Utilizing multi-omics and spatial transcriptomics to develop spatiotemporal maps of immune-endothelial interactions; (2) Investigating the impact of aging and systemic inflammation on the reprogramming of endothelial-immune communication; (3) Innovating nanotherapies for precise delivery across compromised barriers; (4) Establishing validated biomarker panels that integrate neuroimaging with liquid biopsy methodologies. The integration of human organoid models, single-cell technologies, and computational methods will be instrumental in constructing a predictive framework for personalized interventions. Ultimately, targeting the immune-endothelial axis holds significant promise for the development of disease-modifying strategies aimed at maintaining neurovascular homeostasis and achieving sustained neuroprotection in inflammatory neurological disorders.

## Glossary

CNS: central nervous system
BBB: blood-brain barrier
AD: Alzheimer’s disease
MS: multiple sclerosis
ICAM-1: intercellular adhesion molecule-1
VCAM-1: vascular cell adhesion molecule-1
hiPSCs: human induced pluripotent stem cells
BECs: brain endothelial cells
PECAM−1/CD31: platelet endothelial cell adhesion molecule−1
ApoE4: apolipoprotein E ϵ4 allele
TNF−α: tumor necrosis factor−alpha
IL−1β: interleukin−1β
MCAM/CD146: melanoma cell adhesion molecule
EAE: experimental autoimmune encephalomyelitis
ST14: immunoligand tumor suppressor 14
JAM−B: junctional adhesion molecule−B
OSM: oncostatin M
CCL20: C−C motif chemokine ligand 20
IL−17: interleukin−17
IFN−γ: interferon−gamma
MHC: major histocompatibility complex
CXCL12: C−X−C motif chemokine ligand 12
CXCR4: C−X−C chemokine receptor type 4
CSF: cerebrospinal fluid
SASP: senescence−associated secretory phenotype
IL−6: interleukin−6
IL−8: interleukin−8
MCP−1: monocyte chemoattractant protein−1
DAMPs: damage−associated molecular patterns
NLRP3: NLR family pyrin domain containing 3
TBI: traumatic brain injury
ROS: reactive oxygen species
NETs: neutrophil extracellular traps
NK cells: natural killer cells
C3a: complement component 3a
C3aR: C3a receptor
mCRP: monomeric C−reactive protein
EPHA1: EPH receptor A1
HIV: human immunodeficiency virus
P2X7R: purinergic receptor P2X7
ALS: amyotrophic lateral sclerosis
CD39: ectonucleoside triphosphate diphosphohydrolase−1.

## References

[1] Rust R Yin H Achón Buil B Sagare AP Kisler K . The blood-brain barrier: a help and a hindrance. Brain. (2025) 148:2262–82. doi: 10.1093/brain/awaf068. PMID: 39969549 PMC12233556

[2] Sweeney MD Zhao Z Montagne A Nelson AR Zlokovic BV . Blood-brain barrier: from physiology to disease and back. Physiol Rev. (2019) 99:21–78. doi: 10.1152/physrev.00050.2017. PMID: 30280653 PMC6335099

[3] Salvador AFM Abduljawad N Kipnis J . Meningeal lymphatics in central nervous system diseases. Annu Rev Neurosci. (2024) 47:323–44. doi: 10.1146/annurev-neuro-113023-103045. PMID: 38648267 PMC12051392

[4] Shi FD Yong VW . Neuroinflammation across neurological diseases. Science. (2025) 388:eadx0043. doi: 10.1126/science.adx0043. PMID: 40536983

[5] Iadecola C Buckwalter MS Anrather J . Immune responses to stroke: mechanisms, modulation, and therapeutic potential. J Clin Invest. (2020) 130:2777–88. doi: 10.1172/jci135530. PMID: 32391806 PMC7260029

[6] Leng F Edison P . Neuroinflammation and microglial activation in alzheimer disease: where do we go from here? Nat Rev Neurol. (2021) 17:157–72. doi: 10.1038/s41582-020-00435-y. PMID: 33318676

[7] Proulx ST Engelhardt B . Central nervous system zoning: how brain barriers establish subdivisions for cns immune privilege and immune surveillance. J Intern Med. (2022) 292:47–67. doi: 10.1111/joim.13469. PMID: 35184353 PMC9314672

[8] Profaci CP Munji RN Pulido RS Daneman R . The blood-brain barrier in health and disease: important unanswered questions. J Exp Med. (2020) 217:e20190062. doi: 10.1084/jem.20190062. PMID: 32211826 PMC7144528

[9] Nation DA Sweeney MD Montagne A Sagare AP D'Orazio LM Pachicano M . Blood-brain barrier breakdown is an early biomarker of human cognitive dysfunction. Nat Med. (2019) 25:270–6. doi: 10.1038/s41591-018-0297-y. PMID: 30643288 PMC6367058

[10] Ihezie SA Mathew IE McBride DW Dienel A Blackburn SL Thankamani Pandit PK . Epigenetics in blood-brain barrier disruption. Fluids Barr CNS. (2021) 18:17. doi: 10.1186/s12987-021-00250-7. PMID: 33823899 PMC8025355

[11] Fournier AP Tastet O Charabati M Hoornaert C Bourbonnière L Klement W . Single-cell transcriptomics identifies brain endothelium inflammatory networks in experimental autoimmune encephalomyelitis. Neurol Neuroimmunol Neuroinflamm. (2023) 10:e200046. doi: 10.1212/nxi.0000000000200046. PMID: 36446612 PMC9709715

[12] Wälchli T Ghobrial M Schwab M Takada S Zhong H Suntharalingham S . Single-cell atlas of the human brain vasculature across development, adulthood and disease. Nature. (2024) 632:603–13. doi: 10.1038/s41586-024-07493-y. PMID: 38987604 PMC11324530

[13] Nishihara H Perriot S Gastfriend BD Steinfort M Cibien C Soldati S . Intrinsic blood-brain barrier dysfunction contributes to multiple sclerosis pathogenesis. Brain. (2022) 145:4334–48. doi: 10.1093/brain/awac019. PMID: 35085379 PMC10200307

[14] Matsuo K Nagamatsu J Nagata K Umeda R Shiota T Morimoto S . Establishment of a novel amyotrophic lateral sclerosis patient (TARDBP (N345K/+))-derived brain microvascular endothelial cell model reveals defective Wnt/β-catenin signaling: investigating diffusion barrier dysfunction and immune cell interaction. Front Cell Dev Biol. (2024) 12:1357204. doi: 10.3389/fcell.2024.1357204. PMID: 39211392 PMC11357944

[15] Page MJ McKenzie JE Bossuyt PM Boutron I Hoffmann TC Mulrow CD . The prisma 2020 statement: an updated guideline for reporting systematic reviews. Int J Surg. (2021) 88:105906. doi: 10.1016/j.ijsu.2021.105906. PMID: 33789826

[16] Akl EA Khabsa J Iannizzi C Piechotta V Kahale LA Barker JM . Extension of the prisma 2020 statement for living systematic reviews (prisma-lsr): checklist and explanation. Bmj. (2024) 387:e079183. doi: 10.1136/bmj-2024-079183. PMID: 39562017 PMC12036629

[17] Mamuladze T Zaninelli TH Smyth LCD Wu Y Abramishvili D Silva R . Mast cells regulate the brain-dura interface and csf dynamics. Cell. (2025) 188:5487–98:e16. doi: 10.1016/j.cell.2025.06.046. PMID: 40712577 PMC12380238

[18] Huang J Ding J Wang X Gu C He Y Li Y . Transfer of neuron-derived α-synuclein to astrocytes induces neuroinflammation and blood-brain barrier damage after methamphetamine exposure: involving the regulation of nuclear receptor-associated protein 1. Brain Behav Immun. (2022) 106:247–61. doi: 10.1016/j.bbi.2022.09.002. PMID: 36089218

[19] Yang LX Yao YY Yang JR Cheng HL Zhu XJ Zhang ZJ . Sphingosine 1-phosphate receptor 1 regulates blood-brain barrier permeability in epileptic mice. Neural Regener Res. (2023) 18:1763–9. doi: 10.4103/1673-5374.360263. PMID: 36751803 PMC10154506

[20] Winneberger J Schöls S Lessmann K Rández-Garbayo J Bauer AT Mohamud Yusuf A . Platelet endothelial cell adhesion molecule-1 is a gatekeeper of neutrophil transendothelial migration in ischemic stroke. Brain Behav Immun. (2021) 93:277–87. doi: 10.1016/j.bbi.2020.12.026. PMID: 33388423

[21] Zhang Z Gan Q Han J Tao Q Qiu WQ Madri JA . CD31 as a probable responding and gate-keeping protein of the blood-brain barrier and the risk of alzheimer's disease. J Cereb Blood Flow Metab. (2023) 43:1027–41. doi: 10.1177/0271678x231170041. PMID: 37051650 PMC10291450

[22] Fournier AP Zandee S Charabati M Peelen E Tastet O Alvarez JI . CLMP promotes leukocyte migration across brain barriers in multiple sclerosis. Neurol Neuroimmunol Neuroinflamm. (2022) 9:e200022. doi: 10.1212/nxi.0000000000200022. PMID: 36241608 PMC9465835

[23] Zondler L Herich S Kotte P Körner K Schneider-Hohendorf T Wiendl H . MCAM/CD146 signaling via PLCγ1 leads to activation of β(1)-integrins in memory t-cells resulting in increased brain infiltration. Front Immunol. (2020) 11:599936. doi: 10.3389/fimmu.2020.599936. PMID: 33381120 PMC7767877

[24] Charabati M Zandee S Fournier AP Tastet O Thai K Zaminpeyma R . MCAM+ brain endothelial cells contribute to neuroinflammation by recruiting pathogenic CD4+ t lymphocytes. Brain. (2023) 146:1483–95. doi: 10.1093/brain/awac389. PMID: 36319587 PMC10115172

[25] Pareja J Aydin S Zbinden M Bouillet E Zollinger N Theivendram V . Lack of junctional adhesion molecule (JAM)-B traps CD8 t cells in cns border zones and ameliorates autoimmune neuroinflammation. Acta Neuropathol Commun. (2025) 13:117. doi: 10.1186/s40478-025-02021-z. PMID: 40420242 PMC12105289

[26] Zheng X Ren B Gao Y . Tight junction proteins related to blood-brain barrier and their regulatory signaling pathways in ischemic stroke. BioMed Pharmacother. (2023) 165:115272. doi: 10.1016/j.biopha.2023.115272. PMID: 37544283

[27] Greene C Hanley N Campbell M . Blood-brain barrier associated tight junction disruption is a hallmark feature of major psychiatric disorders. Transl Psychiatry. (2020) 10:373. doi: 10.1038/s41398-020-01054-3. PMID: 33139732 PMC7606459

[28] Fetsko AR Sebo DJ Budzynski LB Scharbarth A Taylor MR . IL-1β disrupts the initiation of blood-brain barrier development by inhibiting endothelial Wnt/β-catenin signaling. iScience. (2024) 27:109651. doi: 10.1016/j.isci.2024.109651. PMID: 38638574 PMC11025013

[29] Iliev ID Lin WY Gaffen SL . When IL-17 gets on your nerves. Cell. (2023) 186:466–8. doi: 10.1016/j.cell.2022.12.048. PMID: 36736299 PMC10967262

[30] Hermans D Houben E Baeten P Slaets H Janssens K Hoeks C . Oncostatin M triggers brain inflammation by compromising blood-brain barrier integrity. Acta Neuropathol. (2022) 144:259–81. doi: 10.1007/s00401-022-02445-0. PMID: 35666306

[31] Koehn LM Nguyen KV Tucker R Lim YP Chen X Stonestreet BS . Inter-alpha inhibitor proteins modulate microvascular endothelial components and cytokines after exposure to hypoxia-ischemia in neonatal rats. Mol Neurobiol. (2025) 62:5057–72. doi: 10.1007/s12035-024-04594-7. PMID: 39505805

[32] Mendonça LO Frémond ML . Interferonopathies: from concept to clinical practice. Best Pract Res Clin Rheumatol. (2024) 38:101975. doi: 10.1016/j.berh.2024.101975. PMID: 39122631

[33] Viengkhou B Hayashida E McGlasson S Emelianova K Forbes D Wiseman S . The brain microvasculature is a primary mediator of interferon-α neurotoxicity in human cerebral interferonopathies. Immunity. (2024) 57:1696–709:e10. doi: 10.1016/j.immuni.2024.05.017. PMID: 38878770 PMC11250091

[34] Quan P Li X Si Y Sun L Ding FF Fan Y . Single cell analysis reveals the roles and regulatory mechanisms of type-I interferons in parkinson's disease. Cell Commun Signal. (2024) 22:212. doi: 10.1186/s12964-024-01590-1. PMID: 38566100 PMC10985960

[35] Wang S de Fabritus L Kumar PA Werner Y Ma M Li D . Brain endothelial CXCL12 attracts protective natural killer cells during ischemic stroke. J Neuroinflamm. (2023) 20:8. doi: 10.1186/s12974-023-02689-x. PMID: 36631780 PMC9835334

[36] Li Y Ru X Xu Y Guo P Zhou J Li W . Single-cell sequencing reveals intracranial microvasculature-derived CXCL12 promotes CD8(+) t-cell infiltration and blood-brain barrier dysfunction after subarachnoid hemorrhage in mice. J Neuroinflamm. (2025) 22:116. doi: 10.1186/s12974-025-03444-0. PMID: 40270006 PMC12020083

[37] Aydin S Pareja J Schallenberg VM Klopstein A Gruber T Page N . Antigen recognition detains CD8(+) t cells at the blood-brain barrier and contributes to its breakdown. Nat Commun. (2023) 14:3106. doi: 10.1038/s41467-023-38703-2. PMID: 37253744 PMC10229608

[38] Ali-Moussa S Deczkowska A . Keep your neutrophils close, but your mast cells closer. Trends Immunol. (2025) 46:705–7. doi: 10.1016/j.it.2025.09.005. PMID: 40973531

[39] Pinho-Correia LM McCullough SJC Ghanizada H Nedergaard M Rustenhoven J Da Mesquita S . CSF transport at the brain-meningeal border: effects on neurological health and disease. Lancet Neurol. (2025) 24:535–47. doi: 10.1016/s1474-4422(25)00115-2. PMID: 40409317 PMC12315021

[40] McDonald DM Alitalo K Betsholtz C Engelhardt B Proulx ST Siegenthaler J . Cerebrospinal fluid draining lymphatics in health and disease: advances and controversies. Nat Cardiovasc Res. (2025) 4:1047–65. doi: 10.1038/s44161-025-00705-2. PMID: 40921861

[41] Wang Y Tan Q Pan M Yu J Wu S Tu W . Minimally invasive vagus nerve stimulation modulates mast cell degranulation via the microbiota-gut-brain axis to ameliorate blood-brain barrier and intestinal barrier damage following ischemic stroke. Int Immunopharmacol. (2024) 132:112030. doi: 10.1016/j.intimp.2024.112030. PMID: 38603861

[42] Zheng MY Luo LZ . The role of IL-17A in mediating inflammatory responses and progression of neurodegenerative diseases. Int J Mol Sci. (2025) 26:2505. doi: 10.3390/ijms26062505. PMID: 40141149 PMC11941770

[43] Kothari R Abdulrahim MW Oh HJ Capuzzi DH Kilgore CB Nair SK . A mast cell receptor mediates post-stroke brain inflammation via a dural-brain axis. Cell. (2025) 188:5499–515:e20. doi: 10.1016/j.cell.2025.06.045. PMID: 40712576 PMC12313293

[44] Lohat V Ilyas R Wei Q Oo D Xue J Horsman I . Distinct role of dural and leptomeningeal macrophages in maintaining cerebrospinal fluid drainage to meningeal lymphatic vessels. Am J Pathol. (2025) 195:1660–75. doi: 10.1016/j.ajpath.2025.05.017. PMID: 40541712 PMC12489351

[45] Gonzales MM Garbarino VR Pollet E Palavicini JP Kellogg DL Kraig E . Biological aging processes underlying cognitive decline and neurodegenerative disease. J Clin Invest. (2022) 132:e158453. doi: 10.1172/jci158453. PMID: 35575089 PMC9106343

[46] Santisteban MM Iadecola C . The pathobiology of neurovascular aging. Neuron. (2025) 113:49–70. doi: 10.1016/j.neuron.2024.12.014. PMID: 39788087 PMC12136575

[47] Zhang W Sun HS Wang X Dumont AS Liu Q . Cellular senescence, DNA damage, and neuroinflammation in the aging brain. Trends Neurosci. (2024) 47:461–74. doi: 10.1016/j.tins.2024.04.003. PMID: 38729785

[48] Basisty N Kale A Jeon OH Kuehnemann C Payne T Rao C . A proteomic atlas of senescence-associated secretomes for aging biomarker development. PloS Biol. (2020) 18:e3000599. doi: 10.1371/journal.pbio.3000599. PMID: 31945054 PMC6964821

[49] Yousefzadeh MJ Flores RR Zhu Y Schmiechen ZC Brooks RW Trussoni CE . An aged immune system drives senescence and ageing of solid organs. Nature. (2021) 594:100–5. doi: 10.1038/s41586-021-03547-7. PMID: 33981041 PMC8684299

[50] Matacchione G Borgonetti V Ramini D Silvestrini A Ojetti M Galeotti N . Zingiber officinale Roscoe rhizome extract exerts senomorphic and anti-inflammatory activities on human endothelial cells. Biol (Basel). (2023) 12:438. doi: 10.3390/biology12030438. PMID: 36979130 PMC10045365

[51] Ahire C Nyul-Toth A DelFavero J Gulej R Faakye JA Tarantini S . Accelerated cerebromicrovascular senescence contributes to cognitive decline in a mouse model of paclitaxel (Taxol)-induced chemobrain. Aging Cell. (2023) 22:e13832. doi: 10.1111/acel.13832. PMID: 37243381 PMC10352561

[52] Tarantini S Valcarcel-Ares MN Toth P Yabluchanskiy A Tucsek Z Kiss T . Nicotinamide mononucleotide (NMN) supplementation rescues cerebromicrovascular endothelial function and neurovascular coupling responses and improves cognitive function in aged mice. Redox Biol. (2019) 24:101192. doi: 10.1016/j.redox.2019.101192. PMID: 31015147 PMC6477631

[53] Parodi-Rullán RM Javadov S Fossati S . Dissecting the crosstalk between endothelial mitochondrial damage, vascular inflammation, and neurodegeneration in cerebral amyloid angiopathy and Alzheimer's disease. Cells. (2021) 10:2903. doi: 10.3390/cells10112903. PMID: 34831125 PMC8616424

[54] Zanini G Selleri V Lopez Domenech S Malerba M Nasi M Mattioli AV . Mitochondrial DNA as inflammatory DAMP: a warning of an aging immune system? Biochem Soc Trans. (2023) 51:735–45. doi: 10.1042/bst20221010. PMID: 37013978

[55] Wang QQ Yin G Huang JR Xi SJ Qian F Lee RX . Ionizing radiation-induced brain cell aging and the potential underlying molecular mechanisms. Cells. (2021) 10:3570. doi: 10.3390/cells10123570. PMID: 34944078 PMC8700624

[56] Mu Q Yao K Syeda MZ Wan J Cheng Q You Z . Neutrophil targeting platform reduces neutrophil extracellular traps for improved traumatic brain injury and stroke theranostics. Adv Sci (Weinh). (2024) 11:e2308719. doi: 10.1002/advs.202308719. PMID: 38520727 PMC11151022

[57] Sas AR Carbajal KS Jerome AD Menon R Yoon C Kalinski AL . A new neutrophil subset promotes CNS neuron survival and axon regeneration. Nat Immunol. (2020) 21:1496–505. doi: 10.1038/s41590-020-00813-0. PMID: 33106668 PMC7677206

[58] Chen R Zhang X Gu L Zhu H Zhong Y Ye Y . New insight into neutrophils: a potential therapeutic target for cerebral ischemia. Front Immunol. (2021) 12:692061. doi: 10.3389/fimmu.2021.692061. PMID: 34335600 PMC8317226

[59] Wang Z Chen G . Immune regulation in neurovascular units after traumatic brain injury. Neurobiol Dis. (2023) 179:106060. doi: 10.1016/j.nbd.2023.106060. PMID: 36871640

[60] Xie D Liu H Xu F Su W Ye Q Yu F . IL33 (Interleukin 33)/ST2 (Interleukin 1 receptor-like 1) axis drives protective microglial responses and promotes white matter integrity after stroke. Stroke. (2021) 52:2150–61. doi: 10.1161/strokeaha.120.032444. PMID: 33902297

[61] Wang M Dufort C Du Z Shi R Xu F Huang Z . IL-33/ST2 signaling in monocyte-derived macrophages maintains blood-brain barrier integrity and restricts infarctions early after ischemic stroke. J Neuroinflamm. (2024) 21:274. doi: 10.1186/s12974-024-03264-8. PMID: 39449077 PMC11515348

[62] Akeret K Buzzi RM Thomson BR Schwendinger N Klohs J Schulthess-Lutz N . MyD88-TLR4-dependent choroid plexus activation precedes perilesional inflammation and secondary brain edema in a mouse model of intracerebral hemorrhage. J Neuroinflamm. (2022) 19:290. doi: 10.1186/s12974-022-02641-5. PMID: 36482445 PMC9730653

[63] Lai CC Nelsen B Frias-Anaya E Gallego-Gutierrez H Orecchioni M Herrera V . Neuroinflammation plays a critical role in cerebral cavernous malformation disease. Circ Res. (2022) 131:909–25. doi: 10.1161/circresaha.122.321129. PMID: 36285625 PMC9669201

[64] Zheng RZ Lee KY Qi ZX Wang Z Xu ZY Wu XH . Neuroinflammation following traumatic brain injury: take it seriously or not. Front Immunol. (2022) 13:855701. doi: 10.3389/fimmu.2022.855701. PMID: 35392083 PMC8981520

[65] Li L Peng R Wang C Chen X Gheyret D Guan S . β2 integrin regulates neutrophil trans endothelial migration following traumatic brain injury. Cell Commun Signal. (2025) 23:70. doi: 10.1186/s12964-025-02071-9. PMID: 39923080 PMC11806581

[66] Rundek T Tolea M Ariko T Fagerli EA Camargo CJ . Vascular cognitive impairment (VCI). Neurotherapeutics. (2022) 19:68–88. doi: 10.1007/s13311-021-01170-y. PMID: 34939171 PMC9130444

[67] van Olst L Kamermans A Halters S van der Pol SMA Rodriguez E Verberk IMW . Adaptive immune changes associate with clinical progression of Alzheimer's disease. Mol Neurodegener. (2024) 19:38. doi: 10.1186/s13024-024-00726-8. PMID: 38658964 PMC11044380

[68] Zhao D Wang J Zhang F Wang Q Zang M Niu H . Cerebral glymphatic system: structure, regulation, ageing, and mechanisms of encephalopathy. Ageing Res Rev. (2026) 114:102986. doi: 10.1016/j.arr.2025.102986. PMID: 41360293

[69] Propson NE Gedam M Zheng H . Complement in neurologic disease. Annu Rev Pathol. (2021) 16:277–98. doi: 10.1146/annurev-pathol-031620-113409. PMID: 33234021

[70] Bhatia K Ahmad S Kindelin A Ducruet AF . Complement C3a receptor-mediated vascular dysfunction: a complex interplay between aging and neurodegeneration. J Clin Invest. (2021) 131:e144348. doi: 10.1172/jci144348. PMID: 33393493 PMC7773380

[71] Al-Baradie RS Pu S Liu D Zeinolabediny Y Ferris G Sanfeli C . Monomeric C-reactive protein localized in the cerebral tissue of damaged vascular brain regions is associated with neuro-inflammation and neurodegeneration-an immunohistochemical study. Front Immunol. (2021) 12:644213. doi: 10.3389/fimmu.2021.644213. PMID: 33796111 PMC8007856

[72] Pastorello Y Carare RO Banescu C Potempa L Di Napoli M Slevin M . Monomeric C-reactive protein: a novel biomarker predicting neurodegenerative disease and vascular dysfunction. Brain Pathol. (2023) 33:e13164. doi: 10.1111/bpa.13164. PMID: 37158450 PMC10580018

[73] Owens HA Thorburn LE Walsby E Moon OR Rizkallah P Sherwani S . Alzheimer's disease-associated P460L variant of EphA1 dysregulates receptor activity and blood-brain barrier function. Alzheimers Dement. (2024) 20:2016–33. doi: 10.1002/alz.13603. PMID: 38184788 PMC10984439

[74] Kukanja P Langseth CM Rubio Rodríguez-Kirby LA Agirre E Zheng C Raman A . Cellular architecture of evolving neuroinflammatory lesions and multiple sclerosis pathology. Cell. (2024) 187:1990–2009.e19. doi: 10.1016/j.cell.2024.02.030. PMID: 38513664

[75] Montalban X Lebrun-Frénay C Oh J Arrambide G Moccia M Pia Amato M . Diagnosis of multiple sclerosis: 2024 revisions of the McDonald criteria. Lancet Neurol. (2025) 24:850–65. doi: 10.1016/s1474-4422(25)00270-4. PMID: 40975101

[76] Lerma-Martin C Badia IMP Ramirez Flores RO Sekol P Schäfer PSL Riedl CJ . Cell type mapping reveals tissue niches and interactions in subcortical multiple sclerosis lesions. Nat Neurosci. (2024) 27:2354–65. doi: 10.1038/s41593-024-01796-z. PMID: 39501036 PMC11614744

[77] Zhao Y Chen C Xiao X Fang L Cheng X Chang Y . Teriflunomide promotes blood-brain barrier integrity by upregulating claudin-1 via the Wnt/β-catenin signaling pathway in multiple sclerosis. Mol Neurobiol. (2024) 61:1936–52. doi: 10.1007/s12035-023-03655-7. PMID: 37819429

[78] Hansen CE Kamermans A Mol K Berve K Rodriguez-Mogeda C Fung WK . Inflammation-induced TRPV4 channels exacerbate blood-brain barrier dysfunction in multiple sclerosis. J Neuroinflamm. (2024) 21:72. doi: 10.1186/s12974-024-03069-9. PMID: 38521959 PMC10960997

[79] Johann L Soldati S Müller K Lampe J Marini F Klein M . A20 regulates lymphocyte adhesion in murine neuroinflammation by restricting endothelial ICOSL expression in the CNS. J Clin Invest. (2023) 133:e168314. doi: 10.1172/jci168314. PMID: 37856217 PMC10721159

[80] Candelario-Jalil E Dijkhuizen RM Magnus T . Neuroinflammation, stroke, blood-brain barrier dysfunction, and imaging modalities. Stroke. (2022) 53:1473–86. doi: 10.1161/strokeaha.122.036946. PMID: 35387495 PMC9038693

[81] Younas N Fernandez Flores LC Hopfner F Höglinger GU Zerr I . A new paradigm for diagnosis of neurodegenerative diseases: peripheral exosomes of brain origin. Transl Neurodegener. (2022) 11:28. doi: 10.1186/s40035-022-00301-5. PMID: 35527262 PMC9082915

[82] Shvetcov A Thomson S Cho AN Wilkins HM Reed JH Swerdlow RH . Proteome profiling of cerebrospinal fluid using machine learning shows a unique protein signature associated with APOE4 genotype. Aging Cell. (2025) 24:e14439. doi: 10.1111/acel.14439. PMID: 39722190 PMC11984689

[83] Singh MV Uddin MN Covacevich Vidalle M Sutton KR Boodoo ZD Peterson AN . Non-classical monocyte levels correlate negatively with HIV-associated cerebral small vessel disease and cognitive performance. Front Cell Infect Microbiol. (2024) 14:1405431. doi: 10.3389/fcimb.2024.1405431. PMID: 39507948 PMC11537857

[84] Liu R Du S Zhao L Jain S Sahay K Rizvanov A . Autoreactive lymphocytes in multiple sclerosis: pathogenesis and treatment target. Front Immunol. (2022) 13:996469. doi: 10.3389/fimmu.2022.996469. PMID: 36211343 PMC9539795

[85] Yoon JW Kim KM Cho S Cho MJ Park S Hwang D . Th1-poised naive CD4 T cell subpopulation reflects anti-tumor immunity and autoimmune disease. Nat Commun. (2025) 16:1962. doi: 10.1038/s41467-025-57237-3. PMID: 40000667 PMC11861895

[86] Yeung SS Ho YS Chang RC . The role of meningeal populations of type II innate lymphoid cells in modulating neuroinflammation in neurodegenerative diseases. Exp Mol Med. (2021) 53:1251–67. doi: 10.1038/s12276-021-00660-5. PMID: 34489558 PMC8492689

[87] Zhang YJ Cheng Y Tang HL Yue Q Cai XY Lu ZJ . APOE ϵ4-associated downregulation of the IL-7/IL-7R pathway in effector memory T cells: implications for Alzheimer's disease. Alzheimers Dement. (2024) 20:6441–55. doi: 10.1002/alz.14173. PMID: 39129310 PMC11497660

[88] Zhang Y Wang Y Li Z Wang Z Cheng J Bai X . Vascular-water-exchange MRI (VEXI) enables the detection of subtle AXR alterations in Alzheimer's disease without MRI contrast agent, which may relate to BBB integrity. Neuroimage. (2023) 270:119951. doi: 10.1016/j.neuroimage.2023.119951. PMID: 36805091

[89] Van Camp N Lavisse S Roost P Gubinelli F Hillmer A Boutin H . TSPO imaging in animal models of brain diseases. Eur J Nucl Med Mol Imaging. (2021) 49:77–109. doi: 10.1007/s00259-021-05379-z. PMID: 34245328 PMC8712305

[90] Guilarte TR Rodichkin AN McGlothan JL Acanda De La Rocha AM Azzam DJ . Imaging neuroinflammation with TSPO: a new perspective on the cellular sources and subcellular localization. Pharmacol Ther. (2022) 234:108048. doi: 10.1016/j.pharmthera.2021.108048. PMID: 34848203 PMC9107500

[91] Zhang L Hu K Shao T Hou L Zhang S Ye W . Recent developments on PET radiotracers for TSPO and their applications in neuroimaging. Acta Pharm Sin B. (2021) 11:373–93. doi: 10.1016/j.apsb.2020.08.006. PMID: 33643818 PMC7893127

[92] Cumbers GA Harvey-Latham ED Kassiou M Werry EL Danon JJ . Emerging TSPO-PET radiotracers for imaging neuroinflammation: a critical analysis. Semin Nucl Med. (2024) 54:856–74. doi: 10.1053/j.semnuclmed.2024.09.007. PMID: 39477764

[93] Castillo-González J González-Rey E . Beyond wrecking a wall: revisiting the concept of blood-brain barrier breakdown in ischemic stroke. Neural Regener Res. (2025) 20:1944–56. doi: 10.4103/nrr.Nrr-d-24-00392. PMID: 39254550 PMC11691464

[94] Vollmuth N Sin J Kim BJ . Host-microbe interactions at the blood-brain barrier through the lens of induced pluripotent stem cell-derived brain-like endothelial cells. mBio. (2024) 15:e0286223. doi: 10.1128/mbio.02862-23. PMID: 38193670 PMC10865987

[95] Hajal C Offeddu GS Shin Y Zhang S Morozova O Hickman D . Engineered human blood-brain barrier microfluidic model for vascular permeability analyses. Nat Protoc. (2022) 17:95–128. doi: 10.1038/s41596-021-00635-w. PMID: 34997242

[96] Yan L Moriarty RA Stroka KM . Recent progress and new challenges in modeling of human pluripotent stem cell-derived blood-brain barrier. Theranostics. (2021) 11:10148–70. doi: 10.7150/thno.63195. PMID: 34815809 PMC8581424

[97] Pérez-López A Torres-Suárez AI Martín-Sabroso C Aparicio-Blanco J . An overview of *in vitro* 3D models of the blood-brain barrier as a tool to predict the *in vivo* permeability of nanomedicines. Adv Drug Delivery Rev. (2023) 196:114816. doi: 10.1016/j.addr.2023.114816. PMID: 37003488

[98] Kawakita S Mandal K Mou L Mecwan MM Zhu Y Li S . Organ-on-a-chip models of the blood-brain barrier: recent advances and future prospects. Small. (2022) 18:e2201401. doi: 10.1002/smll.202201401. PMID: 35978444 PMC9529899

[99] Huang B Peng J Huang X Liang F Wang L Shi J . Generation of interconnected neural clusters in multiscale scaffolds from human-induced pluripotent stem cells. ACS Appl Mater Interfaces. (2021) 13:55939–52. doi: 10.1021/acsami.1c18465. PMID: 34788005

[100] Ajongbolo AO Langhans SA . Brain organoids and assembloids-from disease modeling to drug discovery. Cells. (2025) 14:842. doi: 10.3390/cells14110842. PMID: 40498018 PMC12155111

[101] Guzmán-Hernández R Fossati S . Fibrillar tau alters cerebral endothelial cell metabolism, vascular inflammatory activation, and barrier function *in vitro* and *in vivo*. Alzheimers Dement. (2025) 21:e70077. doi: 10.1002/alz.70077. PMID: 40110691 PMC11923556

[102] Jezierski A Huang J Haqqani AS Haukenfrers J Liu Z Baumann E . Mouse embryonic stem cell-derived blood-brain barrier model: applicability to studying antibody triggered receptor mediated transcytosis. Fluids Barr CNS. (2023) 20:36. doi: 10.1186/s12987-023-00437-0. PMID: 37237379 PMC10224255

[103] Chen J Liao S Xiao Z Pan Q Wang X Shen K . The development and improvement of immunodeficient mice and humanized immune system mouse models. Front Immunol. (2022) 13:1007579. doi: 10.3389/fimmu.2022.1007579. PMID: 36341323 PMC9626807

[104] Meng X Yang L Liao Z Sun F Su M Mei Z . Modeling central nervous system disorders in zebrafish: novel insights into pathophysiology and therapeutic discovery. Neurobiol Dis. (2025) 216:107123. doi: 10.1016/j.nbd.2025.107123. PMID: 41015094

[105] Girard SD Julien-Gau I Molino Y Combes BF Greetham L Khrestchatisky M . High and low permeability of human pluripotent stem cell-derived blood-brain barrier models depend on epithelial or endothelial features. FASEB J. (2023) 37:e22770. doi: 10.1096/fj.202201422R. PMID: 36688807

[106] Auer M Bauer A Oftring A Rudzki D Hegen H Bsteh G . Soluble vascular cell adhesion molecule-1 (sVCAM-1) and natalizumab serum concentration as potential biomarkers for pharmacodynamics and treatment response of patients with multiple sclerosis receiving natalizumab. CNS Drugs. (2022) 36:1121–31. doi: 10.1007/s40263-022-00953-x. PMID: 36173556

[107] Clark C Richiardi J Maréchal B Bowman GL Dayon L Popp J . Systemic and central nervous system neuroinflammatory signatures of neuropsychiatric symptoms and related cognitive decline in older people. J Neuroinflamm. (2022) 19:127. doi: 10.1186/s12974-022-02473-3. PMID: 35643540 PMC9148517

[108] Preis L Villringer K Brosseron F Düzel E Jessen F Petzold GC . Assessing blood-brain barrier dysfunction and its association with Alzheimer's pathology, cognitive impairment and neuroinflammation. Alzheimers Res Ther. (2024) 16:172. doi: 10.1186/s13195-024-01529-1. PMID: 39085945 PMC11290219

[109] Cao Y Xu Y Cao M Chen N Zeng Q Lai MKP . Fluid-based biomarkers for neurodegenerative diseases. Ageing Res Rev. (2025) 108:102739. doi: 10.1016/j.arr.2025.102739. PMID: 40122396

[110] Chen S Bao Q Xu W Zhai X . Extracellular particles: emerging insights into central nervous system diseases. J Nanobiotechnol. (2025) 23:263. doi: 10.1186/s12951-025-03354-6. PMID: 40170148 PMC11960037

[111] Heneka MT van der Flier WM Jessen F Hoozemanns J Thal DR Boche D . Neuroinflammation in alzheimer disease. Nat Rev Immunol. (2025) 25:321–52. doi: 10.1038/s41577-024-01104-7. PMID: 39653749 PMC13148177

[112] Orian JM D'Souza CS Kocovski P Krippner G Hale MW Wang X . Platelets in multiple sclerosis: early and central mediators of inflammation and neurodegeneration and attractive targets for molecular imaging and site-directed therapy. Front Immunol. (2021) 12:620963. doi: 10.3389/fimmu.2021.620963. PMID: 33679764 PMC7933211

[113] Shu Y Peng F Zhao B Liu C Li Q Li H . Transfer of patient's peripheral blood mononuclear cells (PBMCs) disrupts blood-brain barrier and induces anti-NMDAR encephalitis: a study of novel humanized PBMC mouse model. J Neuroinflamm. (2023) 20:164. doi: 10.1186/s12974-023-02844-4. PMID: 37443034 PMC10339507

[114] Beard RS Hoettels BA McAllister JM Meegan JE Wertz TS . Progression of experimental autoimmune encephalomyelitis in mice and neutrophil-mediated blood-brain barrier dysfunction requires non-muscle myosin light chain kinase. J Cereb Blood Flow Metab. (2025) 45:1203–20. doi: 10.1177/0271678x251318620. PMID: 39917847 PMC11806455

[115] László MJ Vigh JP Kocsis AE Porkoláb G Hoyk Z Polgár T . N,N-dimethyltryptamine mitigates experimental stroke by stabilizing the blood-brain barrier and reducing neuroinflammation. Sci Adv. (2025) 11:eadx5958. doi: 10.1126/sciadv.adx5958. PMID: 40802766 PMC12346280

[116] Zhang L Pitcher LE Prahalad V Niedernhofer LJ Robbins PD . Targeting cellular senescence with senotherapeutics: senolytics and senomorphics. FEBS J. (2023) 290:1362–83. doi: 10.1111/febs.16350. PMID: 35015337

[117] Saliev T Singh PB . Targeting senescence: a review of senolytics and senomorphics in anti-aging interventions. Biomolecules. (2025) 15:860. doi: 10.3390/biom15060860. PMID: 40563501 PMC12190739

[118] Lee NT Savvidou I Selan C Wright DK Brkljaca R Chia JSJ . Endothelial -targeted CD39 is protective in a mouse model of global forebrain ischaemia. J Neuroinflamm. (2025) 22:115. doi: 10.1186/s12974-025-03394-7. PMID: 40259346 PMC12013200

[119] Xu B Shimauchi-Ohtaki H Yoshimoto Y Sadakata T Ishizaki Y . Transplanted human iPSC-derived vascular endothelial cells promote functional recovery by recruitment of regulatory T cells to ischemic white matter in the brain. J Neuroinflamm. (2023) 20:11. doi: 10.1186/s12974-023-02694-0. PMID: 36650518 PMC9847196

[120] Yue J Tan Y Huan R Guo J Yang S Deng M . Mast cell activation mediates blood-brain barrier impairment and cognitive dysfunction in septic mice in a histamine-dependent pathway. Front Immunol. (2023) 14:1090288. doi: 10.3389/fimmu.2023.1090288. PMID: 36817492 PMC9929573

[121] Boisserand LSB Geraldo LH Bouchart J El Kamouh MR Lee S Sanganahalli BG . VEGF-C prophylaxis favors lymphatic drainage and modulates neuroinflammation in a stroke model. J Exp Med. (2024) 221:e20221983. doi: 10.1084/jem.20221983. PMID: 38442272 PMC10913814

[122] Wei Y Xia X Wang X Yang W He S Wang L . Enhanced BBB penetration and microglia-targeting nanomodulator for the two-pronged modulation of chronically activated microglia-mediated neuroinflammation in Alzheimer's disease. Acta Pharm Sin B. (2025) 15:1098–111. doi: 10.1016/j.apsb.2025.01.015. PMID: 40177541 PMC11959930

[123] Friedman A Prager O Serlin Y Kaufer D . Dynamic modulation of the blood-brain barrier in the healthy brain. Nat Rev Neurosci. (2025) 26:749–64. doi: 10.1038/s41583-025-00976-5. PMID: 41094205

[124] Schindler KA Torices S Schurhoff N Gallo DI Toborek M . The intersection of circadian rhythms and the blood-brain barrier with drug efficacy and delivery in neurological disorders. Adv Drug Delivery Rev. (2025) 224:115645. doi: 10.1016/j.addr.2025.115645. PMID: 40614866 PMC12321092

[125] Fagiani F Di Marino D Romagnoli A Travelli C Voltan D Di Cesare Mannelli L . Molecular regulations of circadian rhythm and implications for physiology and diseases. Signal Transd Targ Ther. (2022) 7:41. doi: 10.1038/s41392-022-00899-y. PMID: 35136018 PMC8825842

[126] Zeng Q Oliva VM Moro M Scheiermann C . Circadian effects on vascular immunopathologies. Circ Res. (2024) 134:791–809. doi: 10.1161/circresaha.123.323619. PMID: 38484032 PMC11867806

[127] Kelters IR Printezi MI Ballesta A Dierickx P Koop Y Innominato PF . Chronotherapy as a novel strategy to limit anthracycline-induced cardiotoxicity. Cardiovasc Res. (2025) 121:2144–56. doi: 10.1093/cvr/cvaf179. PMID: 41052913 PMC12638736

